# Lactate metabolism and protein lactylation in programmed cell death: From novel mechanism to therapeutic strategies in human diseases

**DOI:** 10.1002/ctm2.70795

**Published:** 2026-09-25

**Authors:** Yue Chen, Xiwen Wang, Xiuping Wang, Na Lin, Wenjie Wang, Ruiqi Fu, Yutong Wu, Yi Liu, Hengwei Liu

**Affiliations:** ^1^ Department of Obstetrics and Gynecology Zhongnan Hospital of Wuhan University Wuhan China; ^2^ Department of Gynecology The Affiliated Wuxi People's Hospital of Nanjing Medical University, Wuxi People's Hospital, Wuxi Medical Center, Nanjing Medical University Wuxi China; ^3^ Department of Obstetrics and Gynecology Union Hospital, Tongji Medical College, Huazhong University of Science and Technology Wuhan China

**Keywords:** apoptosis, autophagy, ferroptosis, lactylation, lactate, programmed cell death (PCD), pyroptosis

## Abstract

Protein lactylation is a recently identified post‐translational modification that reflects cellular metabolic status. It plays an important role in regulating gene expression and maintaining essential cellular functions. Dysregulation of lactylation has been implicated in the onset and progression of various human diseases. Programmed cell death (PCD) is a fundamental biological mechanism that determines cellular fate in response to internal and external stress. Although lactylation and PCD were initially regarded as distinct biological processes, recent studies have demonstrated that lactylation directly influences key transcriptional and signaling pathways involved in multiple PCD modalities, thereby reshaping cell‐death regulatory networks under pathological conditions. Nevertheless, the role of lactylation in regulating different forms of PCD across human diseases has not been fully clarified. In this review, we summarize emerging advances that reveal how histone and non‐histone lactylation modulate major PCD pathways and delineate the molecular mechanisms underlying lactylation‐mediated control of cell death programs. Moreover, we discuss the potential of lactylation‐related regulators to serve as promising biomarkers for disease prediction and as attractive targets for therapeutic intervention. Existing studies highlight that lactylation reshapes PCD regulatory circuits under pathological conditions. Lactylation‑associated molecules exhibit great potential as candidate biomarkers for disease prediction and actionable targets for therapeutic intervention. Overall, this review deepens our understanding of how protein lactylation influences programmed cell death and offers new insights into exploiting lactylation‐dependent mechanisms for targeted therapeutic development.

**Key points:**

- Protein lactylation emerges as a key metabolic–epigenetic regulator linking lactate metabolism to programmed cell death.
- Histone and non‐histone lactylation orchestrate apoptosis, pyroptosis, ferroptosis and autophagy across diverse diseases.
- Targeting lactylation‐related pathways provides promising opportunities for disease biomarkers and therapeutic intervention.

## Graphical abstract

This review highlights protein lactylation as a metabolic‐epigenetic regulator linking lactate reprogramming to programmed cell death. By modulating histone and non‐histone targets, lactylation reshapes cell fate decisions and represents a promising biomarker and therapeutic target in human diseases.

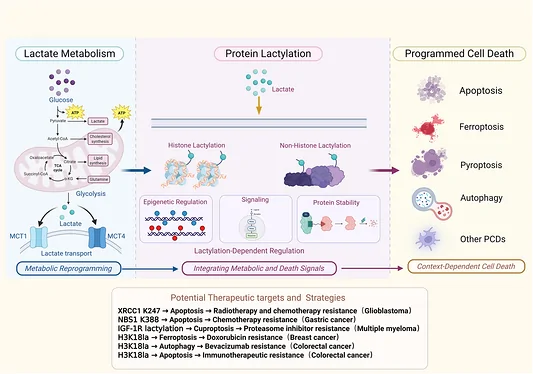

## 1 INTRODUCTION

Protein lactylation is a newly identified post‐translational modification (PTM) derived from cellular lactate metabolism.^1^ Initially discovered as a histone modification influencing gene expression, lactylation has since been recognized as a broader regulatory mechanism affecting multiple biological processes, including immune modulation, metabolic adaptation and cellular homeostasis.^2^ This modification occurs through the addition of lactyl groups to lysine residues, primarily facilitated by acetyltransferases such as p300.^3^ Recent studies suggest that lactylation extends beyond histones to non‐histone proteins, thereby influencing key signaling pathways, including those governing programmed cell death (PCD).^4^, ^5^, ^6^ As a fundamental mechanism for preserving tissue homeostasis and eliminating damaged or abnormal cells, PCD represents a key target for understanding the regulatory impact of lactylation on these pathways,^7^, ^8^ which may provide new insights into disease mechanisms and therapeutic strategies.

PCD encompasses various tightly controlled cellular processes, including apoptosis, necroptosis, pyroptosis and ferroptosis, each playing distinct roles in physiological and pathological contexts.^9^ Apoptosis is a non‐inflammatory form of cell death mediated by the caspase cascade, essential for tissue homeostasis and immune surveillance.^10^, ^11^ In contrast, necroptosis and pyroptosis are lytic forms of cell death associated with inflammatory responses, triggered by receptor‐interacting protein kinases (RIPKs) and inflammasomes, respectively.^12^ Ferroptosis, characterized by iron‐dependent lipid peroxidation, has been implicated in neurodegenerative disorders, cancer and ischaemia–reperfusion injury.^13^ Emerging evidence suggests that lactylation modulates these pathways by regulating key signaling molecules, thereby influencing cell fate decisions. For instance, lactylation may alter the stability or activity of proteins involved in apoptosis, and ferroptosis, thereby affecting disease progression.^14^, ^15^ Exploring the functional implications of lactylation in these pathways may uncover novel regulatory mechanisms linking metabolic shifts to cell death and inflammation.

This review aims to provide a comprehensive overview of the role of lactylation in PCD and its impact on human disease development. We will examine its molecular mechanisms, implications for disease pathogenesis, and potential as a therapeutic target. By integrating current findings, this review seeks to highlight lactylation as a novel regulatory node in cell death pathways, bridging metabolic adaptation and cellular signaling in health and disease.

## 2 LACTATE PRODUCTION, TRANSPORT AND SHUTTLE

### 2.1 Lactate production and clearance

Lactate is produced primarily through glycolysis. Under aerobic conditions, pyruvate enters mitochondria for oxidative phosphorylation; under hypoxia or elevated metabolic demand, pyruvate is reduced to lactate by lactate dehydrogenase (LDH), sustaining ATP production during oxygen deprivation.^16^, ^17^ In tumour cells, glycolysis remains elevated even in the presence of oxygen, a phenomenon known as the Warburg effect, and the resulting lactate surplus supports rapid proliferation.^18^, ^19^

Glutamine, a crucial nitrogen donor and energy intermediate in tumour cells, contributes to lactate production beyond the canonical glycolytic route. Tumour cells convert glutamine to glutamate via glutaminase (GLS), and glutamate is subsequently transformed to alpha‐ketoglutarate (alpha‐KG) by glutamate dehydrogenase (GDH) or transaminase (TA). Within the TCA cycle, alpha‐KG drives ATP synthesis through redox reactions, and glutamine‐derived carbon is progressively metabolized through oxaloacetate to malate. Once translocated to the cytoplasm, malate is converted by malic enzyme (ME1) to pyruvate, which is then reduced to lactate by LDH, establishing a direct link between glutaminolysis and the tumour glycolytic phenotype.^20^, ^21^ The multifaceted roles of lactate in cancer, from metabolic fuel to signalling molecule, have been reviewed in the context of cancer hallmarks.^22^, ^23^, ^24^, ^25^

Although glycolysis and glutaminolysis are generally regarded as the principal metabolic sources of intracellular lactate and protein lactylation, emerging evidence indicates that lactylation is not governed exclusively by these canonical pathways. Extracellular lactate can be imported through monocarboxylate transporters (MCTs), thereby shaping intracellular lactate homeostasis, and lactate can also act as a signalling molecule through receptors such as HCAR1/GPR81.^21^ Beyond substrate availability, lactylation is dynamically regulated by writer enzymes, including p300/CBP, HBO1, AARS1 and KAT2A, as well as erasers such as HDAC1–3, SIRT1 and SIRT3, indicating that this modification is subject to enzymatic control and is not strictly coupled to glycolytic flux.^22^, ^23^, ^24^, ^25^ Notably, S‐D‐lactoylglutathione, an intermediate of the glyoxalase pathway, can induce non‐enzymatic D‐lactylation of lysine residues through S‐to‐N acyl transfer, independently of both lactate concentration and canonical enzymatic catalysis.^26^, ^27^ Moreover, under physiological conditions, the predominant histone lactylation isomer is L‐lactyl‐lysine (Kla), which is derived from glycolysis‐associated L‐lactate, whereas D‐lactylation and Nε‐carboxyethyl‐lysine (Kce) arise primarily from reactive metabolic intermediates when the glyoxalase system is compromised.^28^ Thus, lactylation should be viewed as the integrated output of classical metabolic supply, extracellular lactate transport, enzymatic writing and erasing and non‐enzymatic chemical modification by reactive intermediates, rather than as a simple surrogate of glycolytic activity.

**FIGURE 1:**
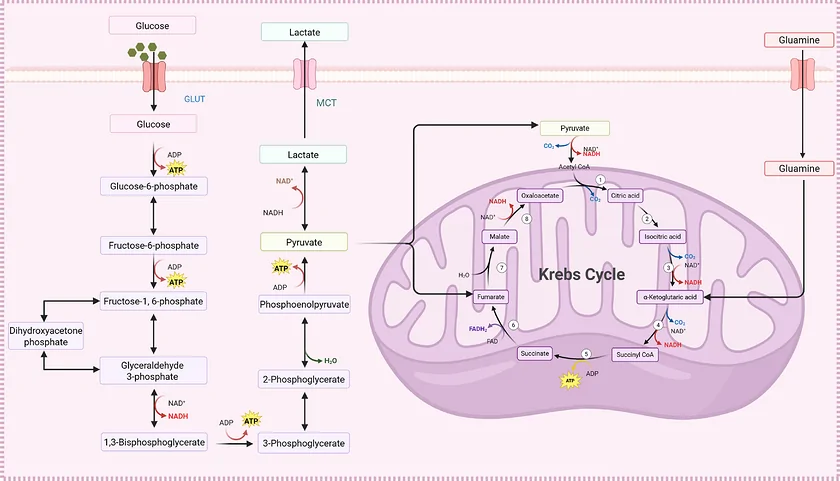
Cellular pathways of lactate generation and utilization. Glucose is taken up via glucose transporters (GLUTs) and metabolized through glycolysis to generate pyruvate, which is either converted to lactate by lactate dehydrogenase or transported into the mitochondria to fuel the tricarboxylic acid (TCA) cycle. Lactate is exported or imported through monocarboxylate transporters (MCTs) and can be reutilized as a metabolic substrate, while glutamine contributes to TCA cycle replenishment via anaplerosis.

Lactate clearance proceeds through two principal routes. Hepatic gluconeogenesis, known as the Cori cycle, recycles lactate into glucose, replenishing systemic glucose reserves for high‐energy‐demand tissues such as the brain, myocardium, and skeletal muscle.^29^ Alternatively, lactate dehydrogenase B (LDHB) converts lactate to pyruvate, which enters mitochondria for oxidation via the pyruvate dehydrogenase complex and the TCA cycle, ensuring irreversible clearance while maximizing energy extraction^1^ (Figure 1).

### 2.2 Lactate transport and shuttle

Lactate is now recognized as both an energy substrate and a signalling molecule involved in metabolic regulation, immune responses and tumour microenvironment modulation.^30^, ^31^ Its transmembrane transport is mediated primarily by monocarboxylate transporters MCT1 and MCT4. The lactate shuttle theory, proposed by Brooks in 1985, describes metabolic coupling between glycolytic cells, which export lactate via MCT4, and oxidative cells, which import it via MCT1 for mitochondrial oxidation.^32^, ^33^

Lactate shuttling occurs at both intercellular and intracellular levels. Intercellularly, glycolytic cells in skeletal muscle, brain and myocardium export lactate via MCT4, while oxidative cells import it via MCT1 for TCA cycle oxidation. Intracellularly, lactate is transported into mitochondria, where LDHB converts it to pyruvate, coupling glycolysis to aerobic respiration.^32^, ^33^

**FIGURE 2:**
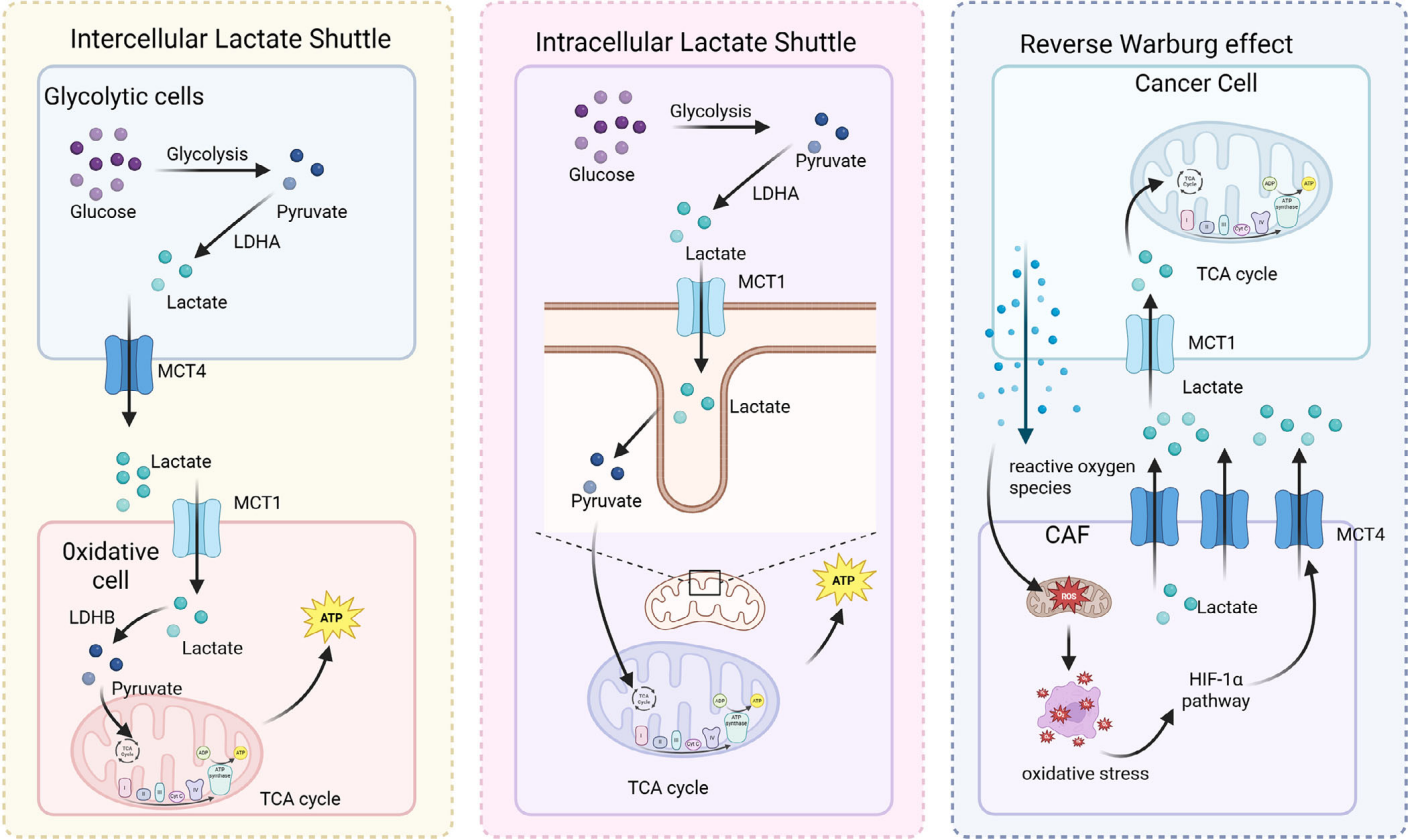
Lactate transport and shuttle. Schematic overview of intercellular and intracellular lactate shuttling, including lactate exchange between glycolytic and oxidative cells, mitochondrial lactate utilization, and lactate‐mediated metabolic coupling in the tumour microenvironment.

In the tumour microenvironment, the lactate shuttle underpins the reverse Warburg effect: cancer‐associated fibroblasts (CAFs) undergo glycolytic reprogramming and export lactate via MCT4, which tumour cells import via MCT1 as metabolic fuel.^34^ A schematic overview of lactate transport and shuttle mechanisms is provided in Figure 2.

### 2.3 Physiological role of lactate

Lactate exerts diverse physiological functions beyond its role as a metabolic intermediate. It participates in systemic energy redistribution: during exercise, lactate produced by fast‐twitch glycolytic muscle fibres is transported through the bloodstream to oxidative tissues including heart, liver and brain, where it serves as an efficient fuel source.^35^, ^36^ In the brain, the astrocyte‐neuron lactate shuttle (ANLS) supplies neurons with astrocyte‐derived lactate to support synaptic activity.^37^ Lactate also functions as a signalling molecule through GPR81/HCAR1, a G‐protein‐coupled receptor that inhibits cyclic AMP (cAMP) production in adipocytes, thereby modulating lipid metabolism. GPR81 expression on immune and endothelial cells further links lactate to inflammatory and vascular regulation.^38^, ^39^, ^40^, ^41^ In the immune system, lactate modulates immune cell polarization and function in a concentration‐ and context‐dependent manner.^42^ Elevated lactate levels in the microenvironment can suppress pro‐inflammatory responses of macrophages and dendritic cells while promoting regulatory T cell (Treg) differentiation.^43^ These effects are particularly evident in the tumour microenvironment, where high lactate concentrations contribute to immune evasion and tumour progression.^44^ Similarly, in inflamed or infected tissues, lactate accumulation serves as a signal to resolve excessive inflammation and initiate tissue repair.^45^ Furthermore, lactate promotes angiogenesis and wound healing by stabilizing HIF‐1alpha and upregulating VEGF, supporting tissue regeneration following ischaemic injury.^46^

## 3 LACTYLATION IS A NEWLY DISCOVERED POST‐TRANSLATIONAL MODIFICATION

### 3.1 The discovery of protein lactylation modification

Protein lactylation, a novel lysine PTM, was independently proposed by two separate research teams in 2019 and 2020, focusing on histones and metabolic enzymes, respectively.^22^, ^26^, ^47^ This discovery marks a new era in lactate metabolism research that bridges epigenetics and signal transduction. The first systematic characterization of this modification was reported by Zhang et al. in *Nature* (2019).^22^ The study aimed to explore whether cellular metabolic state transitions influence transcriptional regulation through specific PTMs. Using lipopolysaccharide (LPS)‐stimulated mouse bone marrow‐derived macrophages (BMDMs) as a model to enhance glycolysis and lactate accumulation, the researchers employed high‐resolution mass spectrometry to identify histone modifications. They detected a novel lysine modification with a mass shift of +72.021 Da, which was later confirmed through isotope labelling and metabolic tracing experiments to originate from intracellular lactate metabolism. This modification, termed ‘lactylation’, was precisely mapped to multiple lysine sites on histone H3 (e.g., H3K18, H3K23). Functional studies revealed that lactylation levels increased with lactate accumulation, particularly during the transition from M1 to M2 macrophages, accompanied by the upregulation of pro‐repair genes (e.g., Arg1). These findings suggest that lactylation plays a role in regulating post‐inflammatory immune state transitions.^22^

In 2020, Gaffney et al. expanded the understanding of lactylation in *Cell Chemical Biology*.^26^ Unlike Zhang's team, which focused on histone‐mediated gene expression regulation, this study investigated the potential lactylation of glycolytic enzymes and explored whether this modification strictly requires enzymatic catalysis. The researchers identified lactoylglutathione (LGSH), an endogenous metabolic intermediate that serves as a lactyl donor for non‐enzymatic acyl transfer reactions with lysine residues. Using stable isotope labelling, liquid chromatography‐mass spectrometry, and synthetic peptide validation, they mapped lactylation sites on key glycolytic enzymes and demonstrated that this modification may regulate enzymatic activity, suggesting a potential negative feedback mechanism in glycolysis.^26^

These two groundbreaking studies have elucidated the mechanisms and biological functions of protein lactylation from distinct perspectives, significantly expanding the scope of PTM research. Furthermore, they have provided novel molecular insights into lactate's roles in critical physiological and pathological processes, including cancer, metabolic disorders and immune responses. This discovery opens new avenues for exploring lactate's multifaceted biological functions within cells and advances our understanding of its complex roles across diverse biological contexts.

### 3.2 Identification of sites involved in protein lactylation modification

Histones are essential structural components of chromatin, responsible for facilitating the tight packaging and structural organization of DNA. In eukaryotic cells, DNA is wound around histone octamers to form nucleosomes, the fundamental units of chromatin. Beyond their architectural role, histones are central to the regulation of gene expression.^48^ They are subject to a variety of epigenetic modifications—including methylation, acetylation, phosphorylation, ubiquitination and lactylation—which dynamically modulate key biological processes such as transcriptional activation, DNA replication, repair and cellular differentiation.^2^ A major breakthrough in the field of histone lactylation was made by Zhang et al., who identified 26 lysine lactylation sites in HeLa cells and 18 sites in mouse bone marrow‐derived macrophages.^22^ Despite recent progress, key questions remain unresolved—such as which chromosomal regions are preferentially marked by histone lactylation and what specific biological processes are regulated by these modifications. Addressing these gaps will require systematic and in‐depth investigations to fully understand the functional implications of histone lactylation.

In recent years, non‐histone lactylation modifications have been consistently identified, demonstrating diverse distributions and modification patterns. Gaffney et al. employed a chemoselective proteomics approach to identify 350 lactylated proteins in HEK293T cells, with the majority of modification sites localized to glycolytic enzymes, suggesting a critical role in energy metabolism regulation.^26^ Concurrently, Hagihara's team observed a significant increase in protein lactylation within the prefrontal cortex of mice under social defeat stress, particularly affecting histone H1. Proteomic analysis revealed 63 lactylated proteins in mouse brain tissue, among which 12 exhibited significantly elevated lactylation levels under stress, implying a potential link between lactylation and neurobehavioral regulation.^49^ Beyond global profiling, studies have begun to unravel the mechanisms of lactylation on individual functional proteins. For instance, Xiong et al. found that lysine residues 281 and 345 of the RNA methyltransferase methyltransferase‐like 3 (METTL3) undergo lactylation in HEK293T cells, enhancing its binding affinity for m6A‐modified RNA and providing molecular insight into lactylation's role in epigenetic regulation.^50^ Taken together, these findings underscore the evolutionary conservation and functional significance of non‐histone protein lactylation in regulating a wide array of physiological and cellular processes.

### 3.3 Protein lactylation modification ‘writers’ and ‘erasers’

Protein lactylation is a reversible post‐translational modification that involves the covalent attachment of a lactyl group to the ε‐amino group of lysine residues. Similar to other types of lysine acylation, this process is dynamically regulated by ‘writers’ that add lactyl groups and ‘erasers’ that remove them. The ‘writers’ of lactylation are primarily lysine acetyltransferases (KATs) that utilize lactyl‐CoA as the donor molecule and transfer lactyl groups to lysine residues on both histone and non‐histone proteins.^22^ Members of the p300/CBP family,^51^, ^52^ as well as KAT8^53^ and TIP60,^54^ have been identified to possess lactyltransferase activity. In addition, enzymes such as AARS1 and AARS2, which are traditionally involved in aminoacyl‐tRNA synthesis, have been reported to catalyze lactylation in cytoplasmic and mitochondrial compartments, respectively.^55^

The intracellular abundance and subcellular distribution of lactyl‐CoA are dynamically regulated, thereby influencing the activity of lactyl‐CoA‐dependent writers. Lactyl‐CoA was first quantified in mammalian cells by Varner et al., who reported concentrations 20‐ to 350‐fold lower than those of acetyl‐CoA.^56^ Two nuclear lactyl‐CoA synthetases have since been identified. ACSS2 converts lactate to lactyl‐CoA and, upon ERK‐mediated phosphorylation at Ser267, translocates to the nucleus where it assembles with the lactyltransferase KAT2A to drive histone H3 lactylation.^56^ GTPSCS represents a second nuclear synthetase that interacts with p300 to facilitate H3K18 lactylation; its nuclear accumulation depends on NLS‐mediated import and acetylation of the G2 subunit at Lys73.^57^ Lactyl‐CoA production is therefore not simply a function of bulk lactate levels. It can be spatially organized at chromatin through signal‐dependent nuclear recruitment of specific synthetases. Beyond enzymatic synthesis, lactyl‐CoA can also form through non‐canonical routes: lactoylglutathione (LGSH) undergoes S‐to‐S acyl transfer to free CoA, generating lactyl‐CoA independently of classical synthetase activity.^58^ Moreover, lactylation does not universally require lactyl‐CoA, as HDAC1‐3 can catalyse lysine lactylation directly using free lactate.^59^ In summary, intracellular lactyl‐CoA is governed by lactate availability, compartment‐specific synthetase activity, modification‐dependent nuclear translocation, and non‐enzymatic thioester chemistry, while a subset of lactylation events proceed through CoA‐independent mechanisms.^60^

Several delactylases have also been identified in recent studies. HDAC1–3, members of class I histone deacetylases, have been shown to exhibit robust delactylase activity toward histone lactylation, thereby modulating gene expression and chromatin structure.^61^ Sirtuin family deacetylases (including SIRT1, SIRT2 and SIRT3), which are NAD^+^‐dependent, also function as delactylases in both nuclear and cytoplasmic contexts. However, despite these advances, the substrate specificity and context‐dependent mechanisms governing lactylation regulation remain unclear, underscoring the need for continued investigation in this field.

### 3.4 Current controversies and technical challenges

Antibody specificity remains a largely underappreciated issue in lysine lactylation detection. Zhang et al. demonstrated that Kla includes three isomeric modifications with identical mass shifts: L‐lactyl‐lysine (Kl‐la), enzymatically formed from glycolysis‐derived L‐lactate; D‐lactyl‐lysine (Kd‐la), generated by non‐enzymatic S‐to‐N acyl transfer from lactoylglutathione (LGSH); and Kce, produced through direct methylglyoxal adduction to lysine residues.^28^ These isomers are not distinguishable by conventional LC–MS/MS, and most commercial pan‐Kla antibodies do not resolve them with sufficient specificity. As a result, earlier proteomic and functional studies based on pan‐Kla antibodies may need to be revisited at isomer‐level resolution. By generating isomer‐specific monoclonal antibodies, this study also provides an important technical foundation for future investigations into lactylation biology.

The enzymatic mechanism of lactylation is also subject to fundamental debate. The modification was initially attributed to p300‐catalyzed transfer using lactyl‐CoA as the donor.^22^ However, Gaffney et al. subsequently found that LGSH can directly lactylate glycolytic enzymes through a non‐enzymatic pathway,^26^ and Trujillo et al. further demonstrated that LGSH can generate lactyl‐CoA non‐enzymatically via S‐S acyl transfer,^58^ indicating that non‐enzymatic routes may contribute substantially to Kla. Two recent studies have proposed alternative, entirely CoA‐independent mechanisms. Zong et al. identified AARS1 and AARS2 as ATP‐dependent lactate sensors and lactyltransferases that use free lactate directly to lactylate substrate proteins^55^; Tsusaka et al. reported that class I histone deacetylases HDAC1/2/3 can reverse their canonical catalytic direction to catalyze lysine lactylation using free lactate as substrate, and that this pathway accounts for the majority of Kla in proliferating cells.^59^ Quantitative data from Varner et al. showed that intracellular lactyl‐CoA concentrations are only 1/20 to 1/350 of acetyl‐CoA levels,^56^ further supporting the predominance of CoA‐independent routes. Collectively, these findings demonstrate that lactylation arises through multiple context‐dependent pathways, and that inferring changes in lactylation solely from modulation of p300 expression or lactate concentration represents an oversimplification of the regulatory mechanisms involved.

At the level of functional validation, most studies to date have inferred the function of lactylation indirectly by manipulating lactate metabolism or p300 activity, without direct site‐specific mutagenesis evidence. Because lactylation and acetylation frequently compete for the same lysine residues, neither K‐R (which mimics the unmodified state) nor K‐Q (which mimics acetylation) can specifically isolate the independent contribution of lactylation. Ren et al. developed a genetic code expansion approach that enables homogeneous incorporation of lactyl‐lysine at defined residues,^62^ providing a methodological basis for precise functional dissection, although the technique has yet to see widespread adoption.

## 4 The Combination of Lactylation and Programmed Cell Death in Human Diseases

As a fundamental process of cells, there are complex connections among multiple PCD, and lactylation is one of the common regulatory nodes in apoptosis, pyroptosis, ferroptosis and autophagy. Lactylation showed similar and/or different roles in multiple PCD. Through epigenetic remodeling, lactylation can influence the transcriptional regulation of PCD‐related genes, modulate the stability of certain key regulatory proteins, and participate in the regulation of mitochondrial function and cellular redox homeostasis, thereby exhibiting shared regulatory features across different types of programmed cell death. Meanwhile, lactylation may exert pathway‐specific effects in distinct PCD modalities by targeting different molecular effectors, contributing to inflammasome activation and amplification of inflammatory cascades, regulation of iron metabolism and lipid peroxidation, or fine‐tuning of autophagy processes and autophagic flux. Overall, these findings provide important clues for understanding the potential common mechanisms and differential roles of lactylation in multiple forms of PCD.

### 4.1 Lactylation modification and pyroptosis

Pyroptosis is a pro‐inflammatory form of PCD mediated by the cleavage of gasdermin D (GSDMD), which forms membrane pores leading to cell lysis and the release of cytokines such as IL‐1β and IL‐18.^63^, ^64^ It can be triggered via two major pathways: the canonical pathway, in which inflammasome activation, including NLRP3 and AIM2, leads to caspase‐1 activation and subsequent cleavage of GSDMD and pro‐inflammatory cytokines^65^, ^66^; and the non‐canonical pathway, in which caspase‐4/5 or caspase‐11 directly sense intracellular LPS and cleave GSDMD independently. The N‐terminal fragment of GSDMD (GSDMD‐N) oligomerizes and inserts into the plasma membrane to execute pyroptosis.^67^ Recent studies have demonstrated that lactylation, a novel metabolic post‐translational modification, may modulate pyroptosis by influencing inflammasome activation and the regulation of pyroptotic regulators.

#### 4.1.1 Histone lactylation and pyroptosis

Histone lactylation has emerged as a key metabolic epigenetic mechanism regulating pyroptosis‐related gene expression across diverse pathological conditions. Notably, H3K18la appears to play a dual role in pyroptosis, depending on the disease context and cellular microenvironment. In neurological disorders, such as bilirubin encephalopathy and ischaemia–reperfusion injury, H3K18la has been shown to promote pyroptosis. Specifically, a recent study showed that in bilirubin encephalopathy, H3K18la was enriched at the NOD2 promoter in astrocytes, upregulating its expression and enhancing MAPK/NF‐κB signalling, which promoted pyroptosis and neuroinflammation.^68^ Similarly, under oxygen–glucose deprivation/reoxygenation (OGD/R) conditions that mimic ischemic injury, lactate accumulation enhances H3K18la deposition at the HMGB1 promoter, which leads to the upregulation of HMGB1, activation of caspase‐1 and GSDMD‐N cleavage, and ultimately and tissue damage.^69^ Another lactylation site, H4K12la, has also been implicated in neurodegeneration. Cheng et al. reported that Aβ stimulation elevates H4K12la levels, which accumulate at the NEK7 promoter to enhance its transcription. Increased NEK7 expression activates caspase‐1, GSDMD‐N, IL‐1β and IL‐18, promoting microglial pyroptosis and aggravating cognitive deficits in an Alzheimer's disease (AD) mouse model.^70^ These findings highlight the role of histone lactylation as a pro‐pyroptotic regulator in the central nervous system. In addition to neurological diseases, histone lactylation also contributes to pyroptosis in occupational diseases. In silicosis, exposure to crystalline silica (CS) induces glycolytic reprogramming and increases lactate production, leading to elevated protein lactylation levels. Lactylation promotes NLRP3 transcription and protein expression, subsequently activating Caspase‐1 and GSDMD which induces macrophage pyroptosis and the release of pro‐inflammatory cytokines.^71^ Interestingly, lactylation may exert context‐dependent effects. In contrast to its pyroptosis‐promoting role in the aforementioned conditions, H3K18la exhibits an anti‐pyroptotic function in inflammatory bowel disease. In a mouse model of ulcerative colitis, H3K18la was found to promote M2 macrophage polarization while suppressing NLRP3 inflammasome activation, thereby alleviating pyroptosis and inflammatory damage.^72^

#### 4.1.2 Non‐histone lactylation and pyroptosis

**FIGURE 3:**
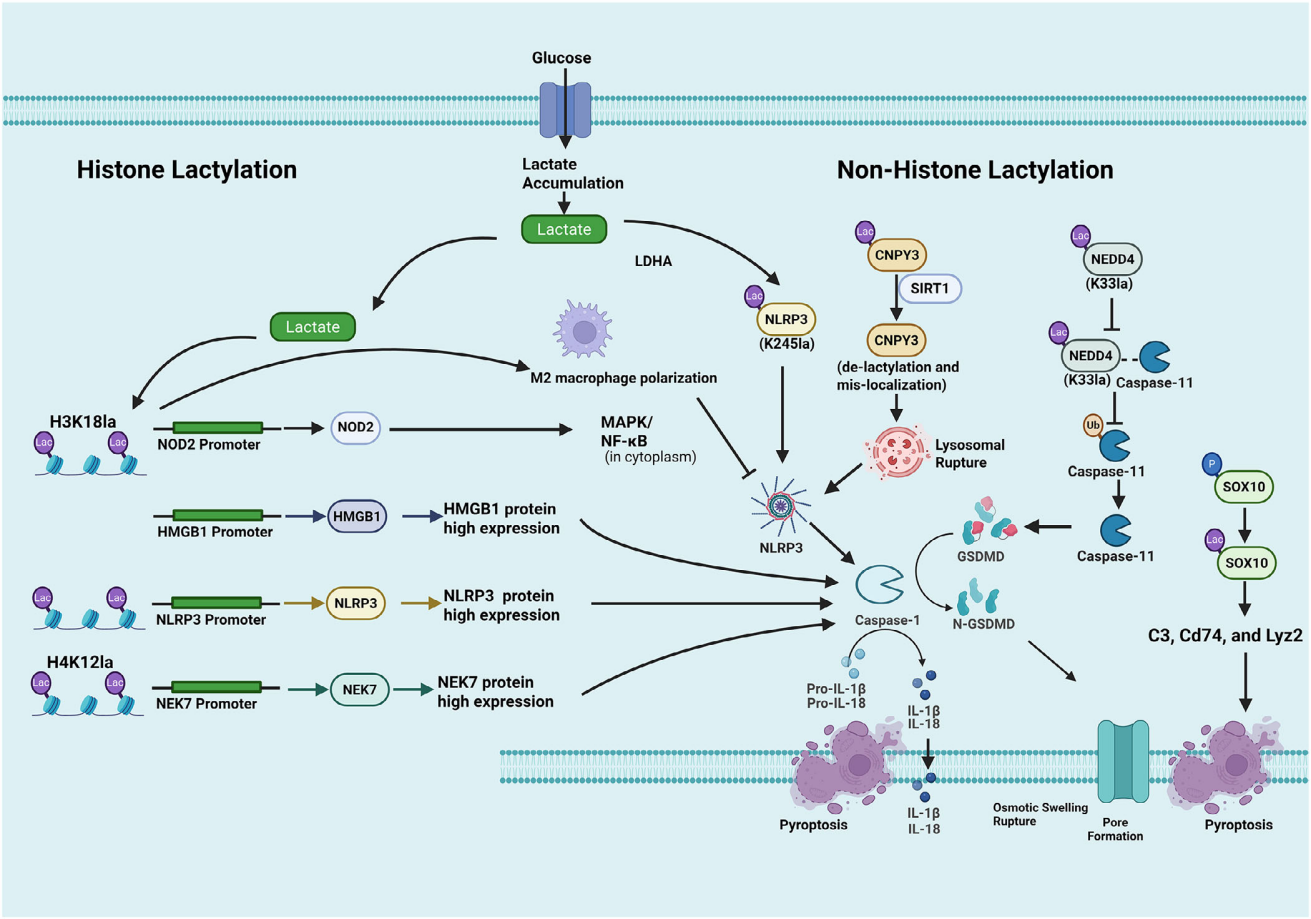
Schematic overview of pyroptosis regulation via lactylation. Lactylation regulates pyroptosis through both histone and non‐histone mechanisms in a context‐dependent manner. Histone lactylation, such as H3K18la and H4K12la, modulates the transcription of pyroptosis‐related genes including NOD2, HMGB1, NEK7, and NLRP3, thereby influencing inflammasome activation, caspase‐1‐mediated GSDMD cleavage, and cytokine release. In parallel, non‐histone lactylation targets key proteins such as NLRP3, CNPY3, NEDD4, Sox10 and caspase‐11, regulating protein stability and inflammasome signaling to control canonical and noncanonical pyroptosis. C3, complement component 3; Caspase‐1, cysteine‐aspartic acid protease‐1; Caspase‐11, cysteine‐aspartic acid protease‐11; Cd74, cluster of differentiation 74; CNPY3, canopy 3; GSDMD, gasdermin D; H3K18la, histone H3 lysine 18 lactylation; H4K12la, histone H4 lysine 12 lactylation; HMGB1, high‐mobility group box 1; IL‐1β, interleukin‐1 beta; IL‐18, interleukin‐18; LDHA, lactate dehydrogenase A; Lyz2, lysozyme 2; MAPK, mitogen‐activated protein kinase; N‐GSDMD, N‐terminal fragment of gasdermin D; NEDD4, neural precursor cell‐expressed developmentally down‐regulated 4; NEK7, NIMA‐related kinase 7; NF‐κB, nuclear factor‐kappa B; NLRP3, NOD‐like receptor family pyrin domain‐containing 3; NOD2, nucleotide‐binding oligomerization domain‐containing 2; SIRT1, silent mating‐type information regulator 1; SOX10, SRY‐box transcription factor 10; Ub, ubiquitin.

Non‐histone lactylation contributes to pyroptosis by stabilizing key inflammasome proteins and altering intracellular signaling. In myocardial ischaemia–reperfusion injury, LDHA‐mediated lactylation of NLRP3 at lysine 245 enhances the stability and expression of this inflammasome component, thereby promoting pyroptosis in cardiomyocytes.^73^ Similarly, in prostate cancer, the interaction between gambogic acid and the chaperone protein CNPY3 recruits the de‐lactylase SIRT1, leading to de‐lactylation and mis‐localization of CNPY3, which causes lysosomal rupture and subsequent activation of the NLRP3/caspase‐1/GSDMD pyroptotic pathway.^74^ Beyond inflammasome stabilization, lactylation also influences pyroptosis through regulation of protein ubiquitination and transcription. For example, in acetaminophen‐induced acute liver injury, lactylation of the E3 ubiquitin ligase NEDD4 at lysine 33 reduces its binding to caspase‐11, thereby decreasing caspase‐11 ubiquitination and increasing its protein stability; under lipopolysaccharide stimulation, this effect synergistically upregulates caspase‐11 expression, triggering noncanonical pyroptosis via GSDMD activation.^75^ In addition, in vascular smooth muscle cells, TNF‐α‐induced phosphorylation of Sox10 primes it for lactylation, which activates transcription of macrophage‐like genes and facilitates pyroptosis, contributing to vascular inflammation.^76^ In asthma, metabolic reprogramming also contributes to lactylation‐mediated pyroptosis. Ovalbumin (OVA) stimulation enhances glycolysis in lung macrophages and THP‐1 cells, increasing intracellular lactate and global protein lactylation. This shift promotes pyroptosis, characterized by elevated pyroptosis‐related proteins.^77^ The mechanism by which lactylation regulates pyroptosis has been partially elucidated. However, further studies are needed to explore the role of lactylation in the regulation of pyroptosis in other diseases (Figure 3).

### 4.2 Lactylation modification and ferroptosis

Ferroptosis is a distinct form of iron‐dependent PCD. It is mainly initiated by the iron‐mediated accumulation of reactive oxygen species (ROS) and a reduced capacity of glutathione peroxidase 4 (GPX4) to eliminate these ROS.^78^ Morphologically, ferroptotic cells are characterized by reduced mitochondrial volume, loss of mitochondrial cristae, and increased membrane density.^78^ Under physiological conditions, polyunsaturated fatty acids (PUFAs) are oxidized by lipoxygenases and subsequently detoxified by GPX4 with the assistance of its key cofactor, glutathione (GSH).^79^ However, in response to pathological stimuli, GPX4 activity declines, leading to weakened cellular antioxidant defence. Consequently, lipid peroxides accumulate and initiate ferroptosis. Simultaneously, intracellular iron levels rise abnormally, and the excess iron generates ROS through the Fenton reaction, further amplifying ferroptosis.^80^ Notably, Emerging evidence suggests that lactylation may regulate ferroptosis by modulating redox homeostasis, iron metabolism, and the expression of ferroptosis‐related genes.

#### 4.2.1 Histone lactylation and ferroptosis

Recent studies have reported that lactylation plays a crucial role in modulating ferroptosis across diverse disease contexts. Key histone lactylation sites, particularly H3K18la regulate the transcription of genes involved in iron metabolism and lipid peroxidation, thereby influencing ferroptotic outcomes. In inflammation‐related diseases, lactylation generally promotes ferroptosis. For example, in sepsis‐induced acute respiratory distress syndrome (ARDS), lactate accumulation elevates H3K14la levels, leading to upregulation of TFRC and downregulation of SLC40A1. This disrupts iron homeostasis, causing intracellular iron overload that enhances reactive oxygen species (ROS) production via the Fenton reaction, exacerbating lipid peroxidation and initiating ferroptosis.^5^ Similarly, in severe acute pancreatitis, elevated H3K18la enhances HIF1A expression and its downstream targets—ACSL4, LPCAT3 and ALOX15—thereby intensifying ferroptosis and disease severity.^81^ In sepsis‐related lung injury, increased H3K18la boosts METTL3 expression, which stabilizes ACSL4 mRNA through YTHDC1 binding, promoting fatty acid metabolism, amplifying ROS production and accelerating ferroptosis.^82^ In contrast, the role of lactylation in tumours is more complex, involving both pro‐ and anti‐ferroptotic effects. In hepatocellular carcinoma, heat stress‐induced lactate accumulation increases H3K18la, which promotes NFS1 transcription and iron‐sulfur cluster biosynthesis, thereby reducing free iron and lipid peroxidation, suppressing ferroptosis, and facilitating tumour cell survival and migration.^83^ In triple‐negative breast cancer, lactate‐induced H3K18la upregulates ZFP64, which transcriptionally activates GCH1 and FTH1 to inhibit lipid peroxidation and iron accumulation, ultimately suppressing ferroptosis.^84^ In prostate cancer, H3K18la enhances HIF1A expression, which promotes lipid accumulation and sensitizes cells to ferroptosis; however, HIF1A also induces GPX4 expression, temporarily suppressing ferroptosis.^85^ Furthermore, in lung cancer, lactylation upregulates AIM2, which destabilizes STAT5B and downregulates ACSL4, thereby inhibiting ferroptosis.^86^ Howerver, it was found that in endometrial cancer, H3K18la promotes p53 expression, which downregulates SLC7A11 and GPX4, enhancing lipid peroxidation and ferroptosis, thus impairing tumour growth and invasion.^6^

#### 4.2.2 Non‐histone lactylation and ferroptosis

**FIGURE 4:**
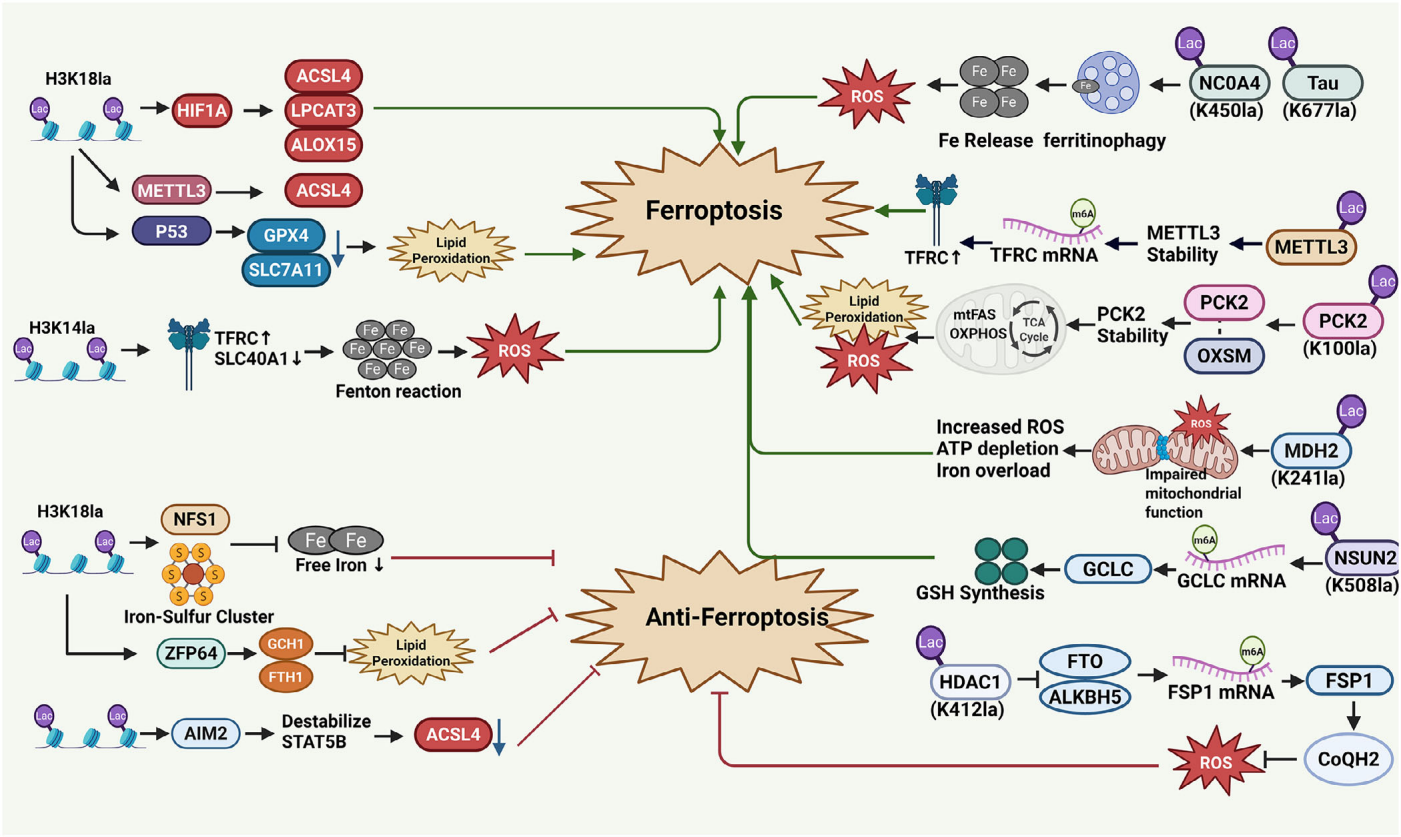
Mechanistic insights into ferroptosis regulation mediated by lactylation. Lactylation regulates ferroptosis through both histone and non‐histone mechanisms by modulating iron metabolism, lipid peroxidation, redox homeostasis and ferroptosis‐related gene expression. Histone lactylation, particularly H3K18la and H3K14la, alters the transcription of key regulators such as HIF1A, ACSL4, METTL3, TFRC, SLC7A11 and GPX4, thereby exerting context‐dependent pro‐ or anti‐ferroptotic effects in inflammatory diseases and cancers. In parallel, non‐histone lactylation directly modifies proteins involved in ferritinophagy, mitochondrial metabolism, RNA methylation and antioxidant defense, including tau, NCOA4, METTL3, PCK2, MDH2, NSUN2 and HDAC1, ultimately influencing ROS accumulation, lipid peroxidation and ferroptotic cell death. ACSL4, acyl‑CoA synthetase long‑chain family member 4; AIM2, absent in melanoma 2; ALOX15, arachidonate‑15‑lipoxygenase; ALKBH5, AlkB homolog 5; CoQH2, ubiquinol (reduced coenzyme Q10); FSP1, ferroptosis suppressor protein 1; FTO, fat mass‑ and obesity‑associated protein; GCLC, glutamate‑cysteine ligase catalytic subunit; GCH1, GTP cyclohydrolase‑1; GPX4, glutathione peroxidase 4; GSH, glutathione; HDAC1, histone deacetylase 1; HIF1A, hypoxia‑inducible factor‑1 alpha; H3K14la, histone H3 lysine 14 lactylation; H3K18la, histone H3 lysine 18 lactylation; LPCAT3, lysophosphatidylcholine acyltransferase 3; MDH2, malate dehydrogenase 2; METTL3, methyltransferase‑like 3; m^6^A, N^6^‑methyladenosine; NCOA4, nuclear receptor coactivator 4; NFS1, NFS1 iron‑sulfur cluster assembly enzyme; NSUN2, NOP2/Sun RNA methyltransferase family member 2; OXSM, 3‑oxoacyl‑ACP synthase, mitochondrial; PCK2, phosphoenolpyruvate carboxykinase 2, mitochondrial; P53, tumour protein p53; ROS, reactive oxygen species; SLC40A1, solute carrier family 40 member 1; SLC7A11, solute carrier family 7 member 11; STAT5B, signal transducer and activator of transcription 5B; TFRC, transferrin receptor; FT(H1), ferritin heavy chain 1; ZFP64, zinc finger protein 64.

In recent years, studies have demonstrated that non‐histone lactylation contributes to the onset and progression of ferroptosis by regulating iron metabolism, mitochondrial function and epigenetic modifications. One key mechanism involves promoting ferritinophagy and disrupting iron homeostasis. In both Alzheimer's disease and cerebral ischaemia, lactylation enhances ferritinophagy, thereby facilitating the release of intracellular iron and promoting ferroptosis. Specifically, lactylation of tau protein at lysine 677 activates the MAPK signaling pathway and upregulates NCOA4 expression, which subsequently promotes ferritinophagy and leads to Fe^2^^+^ accumulation, increased reactive oxygen species (ROS) production and ferroptosis.^87^ Similarly, in cerebral ischaemia models, lactylation of NCOA4 at lysine 450 directly drives ferritinophagy, triggering ferroptosis.^88^ In addition, during intracerebral haemorrhage, elevated lactate levels induce lactylation of METTL3, enhancing its stability and increasing the m^6^A modification of TFRC mRNA. This, in turn, upregulates TFRC expression, promotes iron uptake, and ultimately leads to ferroptosis.^89^ Lactylation also contributes to ferroptosis by impairing mitochondrial function and elevating oxidative stress. During hepatic ischaemia–reperfusion (I/R) injury, lactylation of PCK2 at lysine 100 enhances its interaction with OXSM, preventing its Parkin‐mediated degradation and promoting mitochondrial fatty acid synthesis (mtFAS), the TCA cycle, and oxidative phosphorylation (OXPHOS). This metabolic overactivation leads to ROS accumulation, lipid peroxidation and ferroptosis.^90^ Similarly, in myocardial I/R injury, lactylation of MDH2 at lysine 241 impairs mitochondrial function, characterized by increased ROS, ATP depletion, reduced antioxidant capacity, and iron overload, thereby promoting ferroptosis.^91^ In addition, lactylation influences ferroptosis through transcriptional and epigenetic mechanisms. In the tumour microenvironment, lactate‐induced lactylation of NSUN2 at lysine 508 enhances its m^5^C methyltransferase activity, stabilizing GCLC mRNA and upregulating GCLC protein expression, thereby promoting glutathione synthesis and suppressing ferroptosis.^92^ In colorectal cancer, lactate accumulation drives lactylation of HDAC1 at lysine 412, which downregulates the m^6^A demethylases FTO and ALKBH5, leading to reduced m^6^A demethylation and increased stability of FSP1 mRNA. Elevated FSP1 enhances CoQH_2_ production, which scavenges lipid reactive oxygen species and inhibits ferroptosis. Importantly, HDAC inhibitors decrease HDAC1 lactylation, restore FTO and ALKBH5 levels, destabilize FSP1 mRNA, and sensitize tumour cells to ferroptosis^93^ (Figure 4).

### 4.3 Lactylation modification and autophagy

Autophagy is a conserved, lysosome‐dependent form of PCD that functions primarily to remove and recycle damaged or surplus intracellular organelles and proteins, thereby preserving cellular energy balance and homeostasis.^94^, ^95^ Stress signals such as nutrient deprivation activate the ULK1 complex, initiating autophagy. The Beclin‐1/Vps34 PI3K complex then promotes phagophore formation, which expands with the help of LC3‐II to enclose cytoplasmic cargo, forming a double‐membraned autophagosome. This structure fuses with lysosomes to create autolysosomes, where contents are degraded and recycled.^95^, ^96^, ^97^ Autophagy is regulated by metabolic and epigenetic cues, and recent evidence highlights lactylation as a novel post‐translational modification influencing autophagy by modulating the expression and function of autophagy‐related proteins.

#### 4.3.1 Histone lactylation and autophagy

Histone lactylation exerts diverse regulatory effects on autophagy depending on disease context, specific modification sites, and downstream targets. In tumours, especially colorectal cancer, glioma, and bladder cancer, H3K18la is frequently upregulated and tends to promote autophagy or mitophagy. For instance, H3K18la enhances RUBCNL expression in colorectal cancer, facilitating autophagosome maturation via the PtdIns3K complex.^98^ In glioma, it upregulates YTHDF2, which promotes BNIP3‐dependent mitophagy, thereby supporting metabolic reprogramming, proliferation and apoptosis resistance.^99^ Similarly, in bladder cancer, H3K18la‐driven PRKN expression sustains mitophagy and oxidative metabolism in M2 macrophages, promoting immune evasion and tumour progression.^100^ Beyond tumours, H3K18la also exerts protective effects via mitophagy activation in certain non‐malignant settings. In traumatic brain injury, for example, H3K18la promotes PKM‐mediated activation of the PINK1/PRKN pathway, enhancing mitophagy and supporting neuronal recovery.^101^ In contrast, histone lactylation may act as an autophagy suppressor in some pathological conditions. In hypertrophic scars, increased H3K18la promotes SLUG, which suppresses PTEN transcription, leading to impaired autophagic activity.^102^ In Alzheimer's disease, H4K12la upregulates NLRP3, activating the mTOR pathway and blocking autophagic flux, which contributes to Aβ accumulation and neuroinflammation.^103^ However, histone lactylation does not universally suppress autophagy in AD.^104^ Another study demonstrated that EPB41L4A‐AS1 can regulate lactylation at autophagy‐related gene loci, enhancing Aβ clearance, suggesting a protective, pro‐autophagic effect. This highlights the dual and context‐specific roles of lactylation in neurodegenerative diseases.

#### 4.3.2 Non‐histone lactylation and autophagy

**FIGURE 5:**
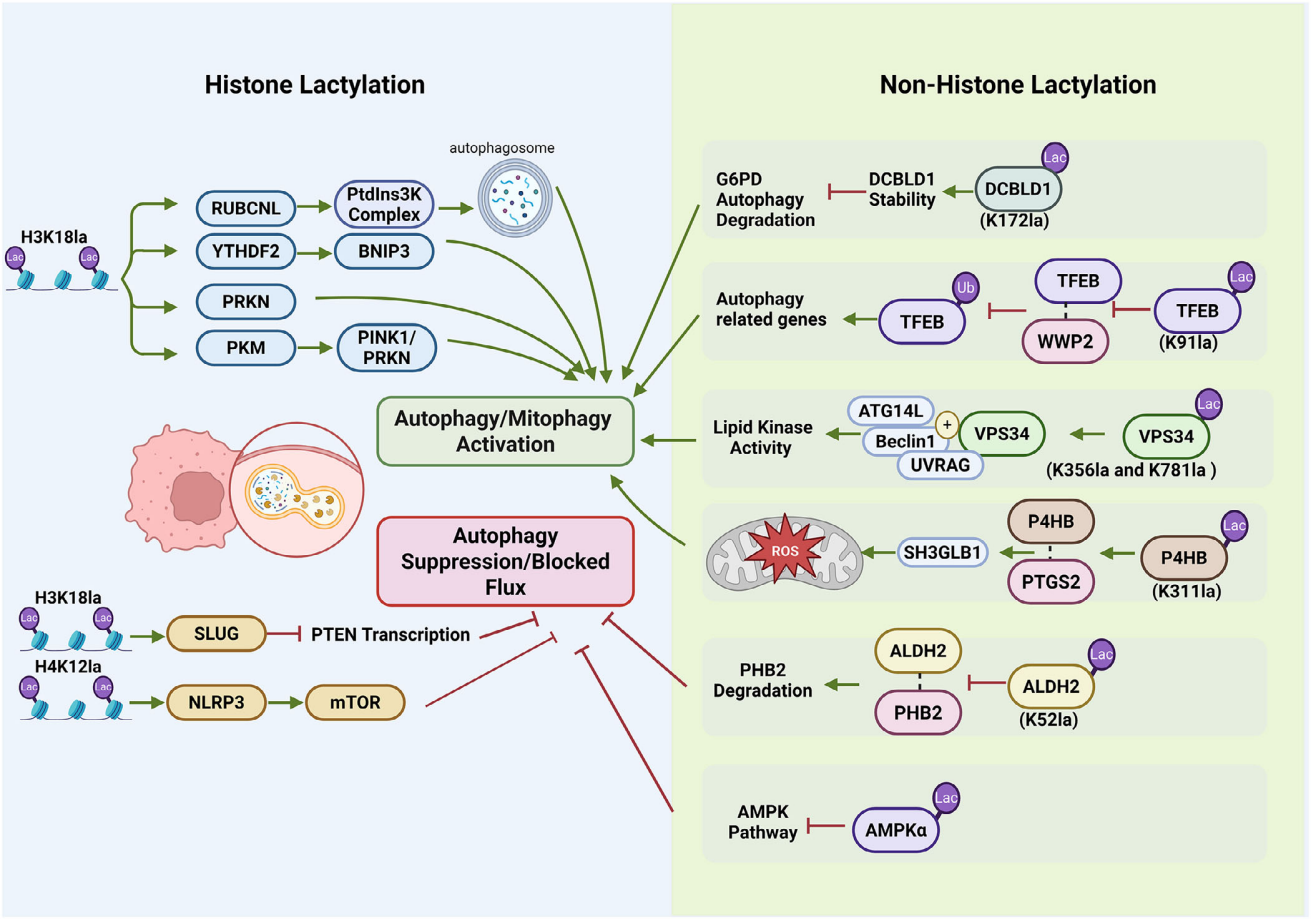
Lactylation impacts autophagic processes through key regulatory nodes. Lactylation modulates autophagy through both histone and non‐histone mechanisms by regulating the transcription and activity of autophagy‐ and mitophagy‐related proteins. Histone lactylation, particularly H3K18la and H4K12la, influences autophagy initiation, autophagosome maturation and mitophagy via transcriptional control of genes involved in the PINK1/PRKN pathway, mTOR signaling, and lysosomal function, exerting context‐dependent pro‐ or anti‐autophagic effects in cancer and neurological disorders. In parallel, non‐histone lactylation directly modifies key regulators such as TFEB, Vps34, ALDH2, AMPKα and P4HB, thereby affecting protein stability, signaling interactions, mitochondrial quality control, and autophagic flux. ALDH2, aldehyde dehydrogenase 2; AMPKα, AMP‐activated protein kinase alpha subunit; ATG14L, autophagy‐related 14‐like; BNIP3, BCL2 interacting protein 3; DCBLD1, discoidin, CUB and LCCL domain containing 1; G6PD, glucose‐6‐phosphate dehydrogenase; H3K18la, histone H3 lysine 18 lactylation; H4K12la, histone H4 lysine 12 lactylation; mTOR, mechanistic target of rapamycin; NLRP3, NLR family pyrin domain containing 3; P4HB, prolyl 4‐hydroxylase subunit beta; PHB2, prohibitin 2; PINK1, PTEN‐induced kinase 1; PKM, pyruvate kinase M1/2; PRKN, parkin RBR E3 ubiquitin protein ligase; PTEN, phosphatase and tensin homolog; PtdIns3K, phosphatidylinositol 3‐kinase; PTGS2, prostaglandin‐endoperoxide synthase 2; ROS, reactive oxygen species; RUBCNL, RUN and cysteine‐rich domain‐containing Beclin 1‐interacting protein‐like; SH3GLB1, SH3 domain‐containing GRB2‐like endophilin B1; SLUG, snail family transcriptional repressor 2; TFEB, transcription factor EB; Ub, ubiquitin; VPS34, vacuolar protein sorting 34; WWP2, WW domain‐containing E3 ubiquitin protein ligase 2; YTHDF2, YTH N6‐methyladenosine RNA‐binding protein 2.

Non‐histone lactylation also plays an important role in the regulation of cellular autophagy. It stabilizes autophagy‐related proteins, thereby promoting tumour progression or enhancing autophagy. In cervical cancer, DCBLD1 lactylation at K172 stabilized the protein, suppressed autophagic degradation of G6PD, and activated the pentose phosphate pathway to enhance tumour progression.^105^ In pancreatic cancer, lactylation of TFEB at lysine 91 disrupts its interaction with the E3 ubiquitin ligase WWP2, thereby preventing TFEB ubiquitination and subsequent proteasomal degradation. The resulting stabilization of TFEB allows its translocation into the nucleus, where it binds to the promoters of autophagy‐ and lysosome‐related genes, enhancing their transcription.^106^ Lactylation also modulates autophagy by influencing interactions among autophagy‐related proteins. Lactate‐driven lactylation of Vps34 at K356 and K781 enhances its lipid kinase activity by strengthening its interaction with Beclin1, ATG14L and UVRAG. This interaction promote autophagy in both cancer and muscle tissues.^107^ In AKI, lactylation of ALDH2 at K52 disrupts its interaction with PHB2, leading to PHB2 degradation and inhibition of mitophagy, thereby exacerbating mitochondrial dysfunction and worsening AKI progression.^108^ Upon X‐ray irradiation, P4HB undergoes lactylation at the K311 site, which enhances its interaction with PTGS2. This promotes SH3GLB1‐mediated accumulation of mitochondrial ROS (mitoROS) and activates NDP52‐induced autophagy, ultimately exacerbating cardiac injury.^109^ Moreover, lactylation regulates autophagy via signaling pathways. In intervertebral disc degeneration, TNF‐α stimulation elevates AMPKα lactylation, which inhibits its phosphorylation, inactivates the AMPK pathway, and consequently impairs matrix synthesis, suppresses autophagy and accelerates senescence^110^ (Figure 5).

### 4.4 Lactylation modification and apoptosis

Apoptosis refers to a controlled, active form of cell death regulated by intrinsic genetic mechanisms, occurring under specific physiological or pathological conditions.^111^ It can be triggered via both intrinsic and extrinsic pathways. The intrinsic pathway, activated by intracellular stress, such as DNA damage, hypoxia, triggers Bcl‐2 family‐mediated mitochondrial outer membrane permeabilization (MOMP), releasing cytochrome C into the cytoplasm. Cytochrome C then forms the apoptosome with Apaf‐1 and procaspase‐9, leading to caspase‐9 activation and downstream cleavage of effector caspases (caspase‐3, ‐6, ‐7), executing apoptosis.^112^ The extrinsic pathway is initiated by ligand binding (e.g., FasL, TNF‐α) to death receptors, activating caspase‐8 or ‐10, which in turn activate effector caspases.^113^ Here, we summarize recent findings on how protein lactylation regulate apoptosis in diverse human diseases.

#### 4.4.1 Histone lactylation and apoptosis

It has been repored that histone lactylation has dual regulatory effects on apoptosis. In neurological, inflammatory, and cardiovascular diseases, histone lactylation often promotes apoptosis. For example, in ischemic stroke, elevated pan‐Kla and H3K18la enhance Apaf‐1 transcription, suppress Bcl‐2, and upregulate Bax and Caspase‐3, thereby triggering neuronal apoptosis.^114^ In schizophrenia, increased H3K9la, and H3K18la promote HMGB1‐mediated neuronal apoptosis.^115^ In sepsis‐associated AKI, H3K18la activates the RhoA/ROCK/Ezrin pathway, leading to NF‐κB activation and increased expression of BAX and decreased expression of BCL2. Lactylation of Ezrin at K263 further amplifies this pro‐inflammatory and pro‐apoptotic response.^14^ Similarly, in aortic aneurysm/dissection, H3K18la upregulates P53, activating downstream Bax and cleaved‐caspase3, and promoting vascular smooth muscle cell apoptosis.^116^ In ischaemia–reperfusion injury, H3K18la promotes the expression of YTHDF2, which, through its intrinsic disordered region positively regulates G3BP1. When YTHDF2 expression is upregulated, it promotes cardiomyocyte apoptosis under OGD/R stimulation.^117^ In contrast, in various tumours, histone lactylation generally inhibits apoptosis and supports tumour progression. In TNBC, H4K12la facilitates binding to the SLFN5 promoter, repressing its transcription and reducing bax and cleaved‐caspase3 expression.^118^ In endometrial cancer, H3K18la upregulates USP39, which stabilizes PGK1 via deubiquitination, activating the PI3K/AKT pathway to suppress apoptosis and enhance cell proliferation.^115^, ^116^, ^119^ Consistently, in liver cancer, elevated levels of H3K9la, H3K14la, and H3K56la are associated with increased expression of the BCL2 and decreased expression of BAX and Caspase‐8, collectively contributing to the suppression of apoptosis and the promotion of tumour cell survival.^120^, ^121^ Furthermore, in lung adenocarcinoma, increased H3K14la and H3K18la at the SLC25A29 promoter suppress its expression, leading to reduced endothelial apoptosis and altered angiogenesis.^122^ Moreover, H3K18la induces lactylation of IDH3G, which enhances tumour cell proliferation and migration while inhibiting apoptosis.^123^ However, a study in colorectal cancer showed an opposite pattern, where H3K18la promoted the transcription of CircATXN7 and inhibited NF‐κB signaling, resulting in decreased expression of anti‐apoptotic genes such as Bcl2, Bcl2l1, Ier3 and Gadd45b, thereby promoting apoptosis. This suggests that histone lactylation may exhibit tumour‐suppressive roles under certain contexts.^124^

#### 4.4.2 Non‐histone lactylation and apoptosis

**FIGURE 6:**
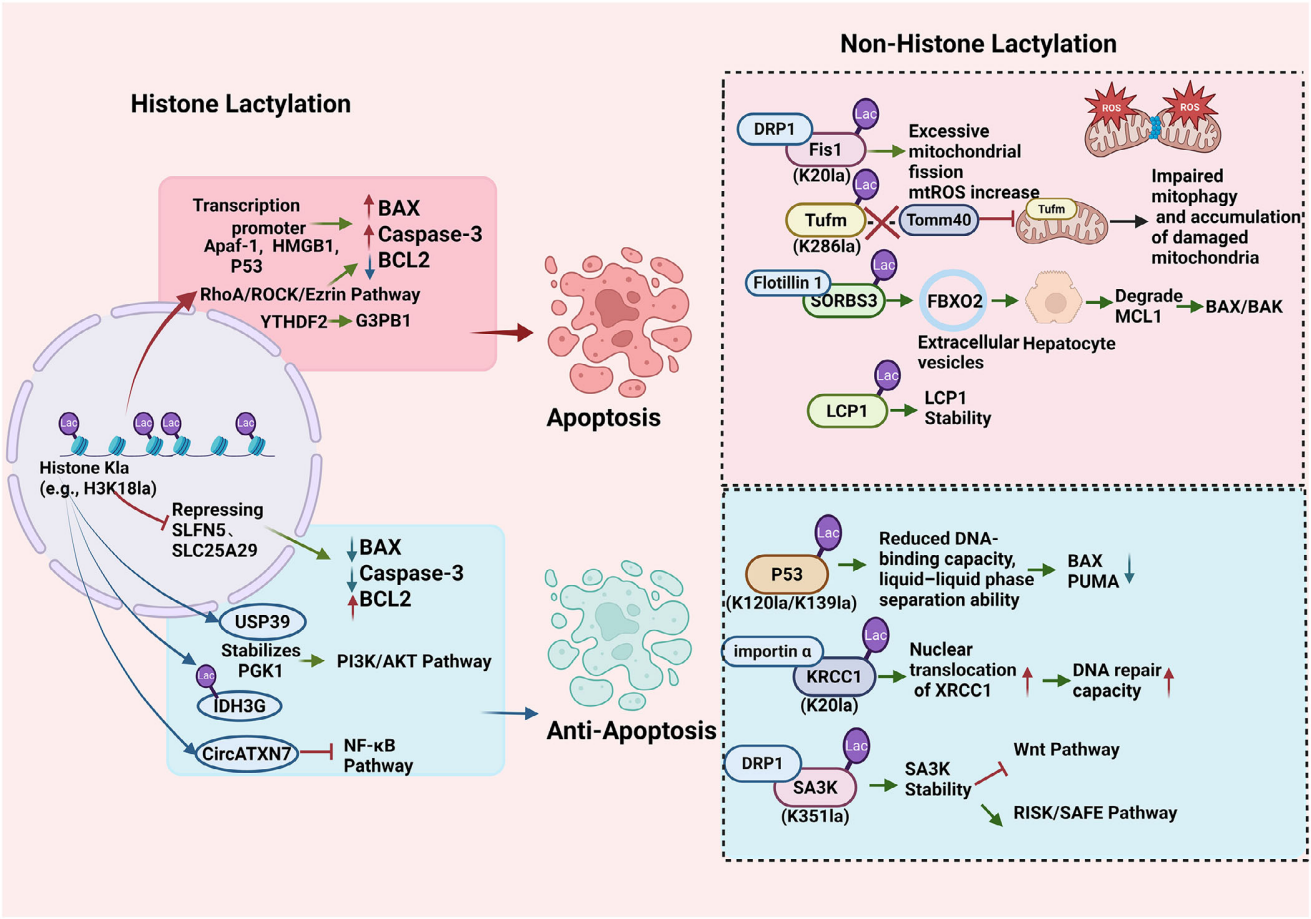
Regulatory role of lactylation in apoptosis modulation. Lactylation regulates apoptosis through both histone and non‐histone mechanisms by modulating the transcription and function of key apoptotic regulators. Histone lactylation, particularly H3K18la and H4K12la, influences the expression of apoptosis‐related genes involved in mitochondrial integrity, caspase activation and survival signaling, exerting context‐dependent pro‐ or anti‐apoptotic effects in neurological, inflammatory, cardiovascular diseases and cancers. In parallel, non‐histone lactylation directly modifies proteins governing mitochondrial dynamics, protein stability, DNA repair and signaling pathways, thereby regulating apoptotic sensitivity and cell fate decisions. AKT, protein kinase B; Apaf‐1, apoptotic protease activating factor 1; BAX, BCL2‐associated X protein; BCL2, B‐cell lymphoma 2; CircATXN7, circular RNA derived from ataxin 7; DRP1, dynamin‐related protein 1; FBXO2, F‐box protein 2; FIS1, mitochondrial fission 1 protein; G3BP1, G3BP stress granule assembly factor 1; H3K18la, histone H3 lysine 18 lactylation; HMGB1, high mobility group box 1; IDH3G, isocitrate dehydrogenase 3 gamma; Kla, lysine lactylation; LCP1, lymphocyte cytosolic protein 1; MCL1, myeloid cell leukemia 1; mtROS, mitochondrial reactive oxygen species; NF‐κB, nuclear factor kappa B; P53, tumour protein p53; PGK1, phosphoglycerate kinase 1; PI3K, phosphoinositide 3‐kinase; PUMA, p53 upregulated modulator of apoptosis; RhoA, Ras homolog family member A; RISK, reperfusion injury salvage kinase; ROCK, Rho‐associated coiled‐coil‐containing protein kinase; SA3K, serine protease inhibitor A3K; SAFE, survivor activating factor enhancement; SLC25A29, solute carrier family 25 member 29; SLFN5, schlafen family member 5; SORBS3, sorbin and SH3 domain containing 3; TOMM40, translocase of outer mitochondrial membrane 40; TUFM, Tu translation elongation factor, mitochondrial; USP39, ubiquitin‐specific peptidase 39; Wnt, Wingless/Int‐1 signaling pathway; XRCC1, X‐ray repair cross‐complementing protein 1; YTHDF2, YTH N6‐methyladenosine RNA‐binding protein 2.

Lactylation also occurs on non‐histone proteins, directly modifying their function and stability to regulate apoptosis. Lactylation modulates apoptosis primarily by regulating protein–protein interactions, influencing cellular processes through the promotion or inhibition of binding between key proteins. In sepsis‐induced acute kidney injury, elevated lactate levels promote the lactylation of Fis1 at the K20 site (Fis1 K20la), which enhances its interaction with DRP1, driving excessive mitochondrial fission. This abnormal fission depletes ATP, increases mitochondrial reactive oxygen species (mtROS) production, ultimately exacerbating cell apoptosis and worsening kidney injury.^125^ In traumatic brain injury, lactylation of Tufm at K286 disrupts its interaction with Tomm40, blocking mitochondrial translocation and impairing mitophagy. This leads to accumulation of damaged mitochondria, triggering inflammation, oxidative stress and neuronal apoptosis.^126^ In exercise‐induced liver fibrosis, lactylation and phase separation of SORBS3 enhance its interaction with Flotillin 1, promoting FBXO2 packaging into extracellular vesicles. FBXO2 enters hepatocytes, degrades MCL1, activates the BAX/BAK pathway, and induces apoptosis, driving fibrosis progression.^127^ Lactylation can also affect protein function by regulating protein stability and degradation. In hepatocellular carcinoma (HCC), reduced expression of SIRT3 leads to lactylation of CCNE2 at the K347 and K348 sites. The lactylation of CCNE2 promotes HCC cell proliferation, migration and invasion, while inhibiting apoptosis.^128^ Lactylation of p53 at K120 and K139 reduces its DNA‐binding and LLPS capacity, downregulates pro‐apoptotic genes like BAX and PUMA, and suppresses apoptosis.^55^ In glioblastoma (GBM), XRCC1 undergoes lactylation at lysine 247, which enhances its affinity for importin α and promotes its nuclear translocation. Once in the nucleus, XRCC1 strengthens DNA repair capacity, leading to resistance to radiotherapy and chemotherapy, and suppresses apoptosis in tumour cells.^129^ In brain infarction‐related studies, OGD/R stimulation promotes glycolysis in PC12 cells, increasing lactate production and overall lactylation, including LCP1. This lactylation enhances LCP1 stability, leading to reduced cell viability and increased apoptosis in PC12 cells.^130^ In cardiac ischaemia–reperfusion injury, lactylation of SA3K at the K351 site enhances its stability, and it is secreted by fibroblasts (FBs) to the extracellular space, where it acts on cardiomyocytes (CMs). On one hand, SA3K inhibits the WNT pathway in CMs, reducing pro‐apoptotic signals; on the other hand, it activates anti‐apoptotic pathways such as RISK and SAFE in CMs^131^ (Figure 6).

### 4.5 Lactylation modification and other PCDs

**FIGURE 7:**
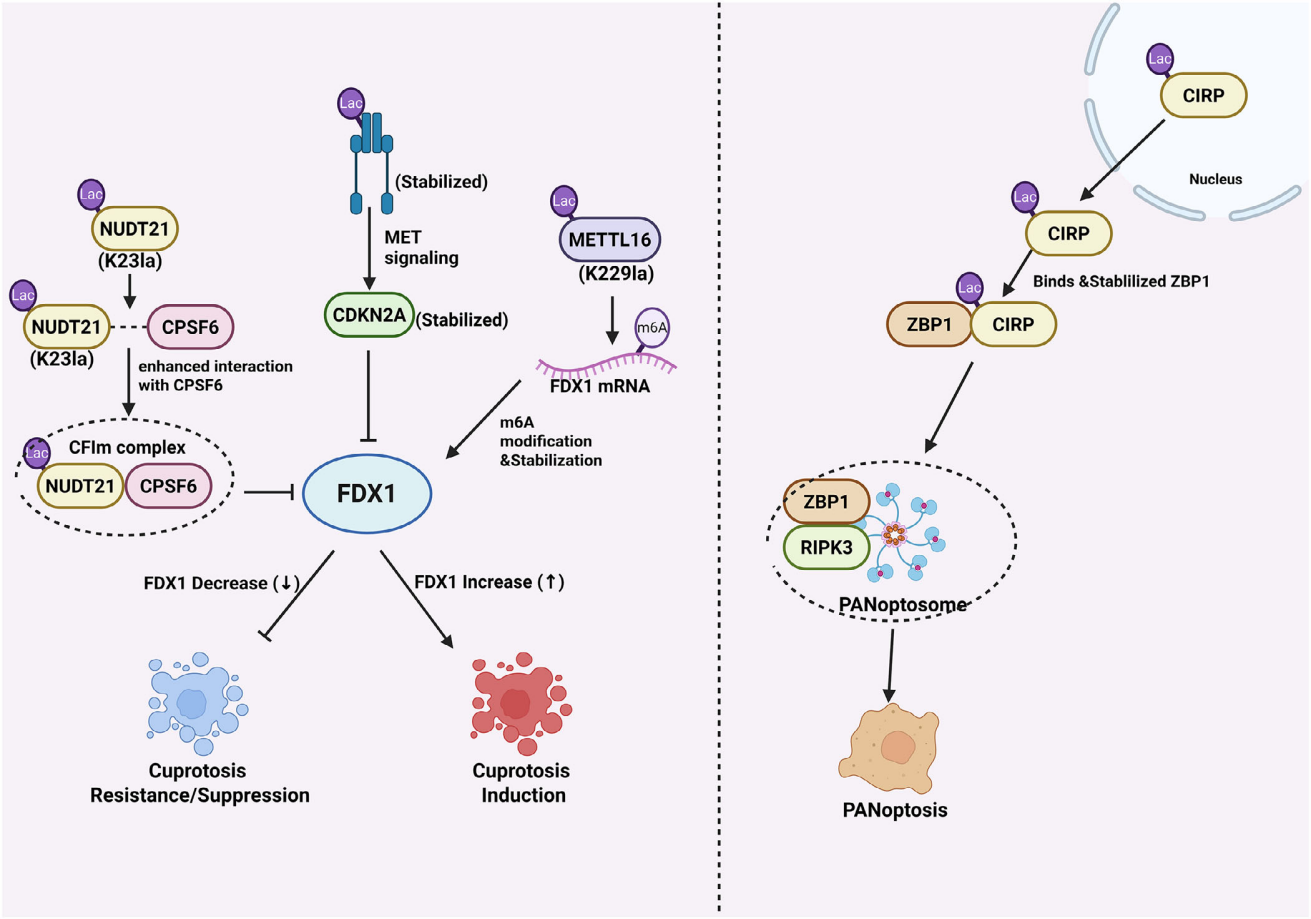
Emerging roles of lactylation in the regulation of other PCD forms. Lactylation modulates additional forms of programmed cell death, including cuproptosis and PANoptosis. By regulating the stability, interaction and epigenetic control of key factors such as FDX1 and PANoptosome‐associated proteins, lactylation may either promote or suppress these cell death pathways across different disease settings. CDKN2A, cyclin‐dependent kinase inhibitor 2A; CFIm, cleavage factor Im; CIRP, cold‐inducible RNA‐binding protein; CPSF6, cleavage and polyadenylation specific factor 6; FDX1, ferredoxin 1; m^6^A, N^6^‐methyladenosine; MET, MET proto‐oncogene receptor tyrosine kinase; METTL16, methyltransferase‐like 16; NUDT21, Nudix hydrolase 21; RIPK3, receptor‐interacting serine/threonine‐protein kinase 3; ZBP1, Z‐DNA‐binding protein 1.

Lactylation also regulates other types of programmed cell death, such as cuproptosis and PANoptosis, in a context‐dependent manner. In esophageal squamous cell carcinoma, lactylation at NUDT21 K23 enhances its interaction with CPSF6, promoting CFIm complex formation and reducing FDX1 expression, thereby conferring resistance to cuproptosis.^132^ In multiple myeloma, IGF‐1R lactylation increases its stability and activates MET signaling, which in turn stabilizes CDKN2A and inhibits FDX1 expression, suppressing cuproptosis.^133^ Conversely, in gastric cancer, lactylation at METTL16 K229 relieves its autoinhibition, promotes m^6^A modification of FDX1 mRNA, enhances its stability, and induces cuproptosis.^134^ Moreover, in sepsis, lactylation facilitates CIRP translocation and extracellular release, enabling it to bind and stabilize ZBP1, which forms the PANoptosome with RIPK3 and activates PANoptosis, aggravating acute lung injury^135^ (Figure 7). Comprehensive surveys of the broader PCD landscape exist,^136^ but the review by Dai et al. predated the discovery of lactylation. Lv et al. examined lactylation through the lens of cancer hallmarks without treating PCD as an independent axis.The molecular mechanisms of lactylation‑mediated programmed cell death are summarized in Table 1, which classifies published findings into several mechanistic categories with detailed information on modification sites, PCD types, disease contexts and functional consequences.^20^

**TABLE 1:** Mechanistic classification of lactylation‐regulated programmed cell death.

Mechanism	Target (Site)	Type	PCD	Disease Context	Functional Consequence	PMID
Transcriptional regulation	H3K18la at NOD2 promoter	Histone	Pyroptosis	Bilirubin encephalopathy	Promotes astrocyte pyroptosis via MAPK/NF‐kappaB	40075479
Transcriptional regulation	H3K18la at HMGB1 promoter	Histone	Pyroptosis	Ischaemia–reperfusion injury	Promotes pyroptosis via HMGB1 upregulation	36870018
Transcriptional regulation	H4K12la at NEK7 promoter	Histone	Pyroptosis	Alzheimer disease	Promotes microglial pyroptosis	39563448
Transcriptional regulation	H3K18la at NLRP3 promoter	Histone	Pyroptosis	Silicosis	Promotes macrophage pyroptosis via caspase‐1/GSDMD	39217895
Transcriptional regulation	H3K18la (anti‐inflammatory)	Histone	Pyroptosis	Ulcerative colitis	Suppresses NLRP3 inflammasome and pyroptosis	34899735
Transcriptional regulation	H3K14la at TFRC/SLC40A1	Histone	Ferroptosis	Sepsis‐induced ARDS	Disrupts iron homeostasis, promotes ferroptosis	39822760
Transcriptional regulation	H3K18la at HIF1A/ACSL4	Histone	Ferroptosis	Severe acute pancreatitis	Enhances lipid peroxidation and ferroptosis	39676583
Transcriptional regulation	H3K18la at METTL3/ACSL4	Histone	Ferroptosis	Sepsis‐associated lung injury	Promotes ferroptosis via m6A modification	38852200
Transcriptional regulation	H3K18la at NFS1 promoter	Histone	Ferroptosis	Hepatocellular carcinoma	Suppresses ferroptosis, promotes metastasis	39970777
Transcriptional regulation	H3K18la at ZFP64/GCH1/FTH1	Histone	Ferroptosis	Triple‐negative breast cancer	Suppresses ferroptosis, doxorubicin resistance	40022222
Transcriptional regulation	H3K18la at RUBCNL	Histone	Autophagy	Colorectal cancer	Promotes autophagosome maturation, bevacizumab resistance	37615625
Transcriptional regulation	H4K12la at SLFN5	Histone	Apoptosis	Triple‐negative breast cancer	Suppresses Bax/caspase‐3, inhibits apoptosis	39395526
Transcriptional regulation	H3K18la at USP39/PGK1	Histone	Apoptosis	Endometrial carcinoma	Activates PI3K/AKT, suppresses apoptosis	38454079
Protein stability (ubiquitination antagonism)	NLRP3 K245la	Non‐histone	Pyroptosis	Myocardial I/R injury	Enhances NLRP3 stability, promotes cardiomyocyte pyroptosis	39548367
Protein stability (ubiquitination antagonism)	NEDD4 K33la	Non‐histone	Pyroptosis	APAP‐induced liver injury	Reduces caspase‐11 ubiquitination, promotes non‐canonical pyroptosis	38412862
Protein stability (ubiquitination antagonism)	TFEB K91la	Non‐histone	Autophagy	Pancreatic ductal adenocarcinoma	Blocks WWP2 binding, stabilizes TFEB, enhances autophagy	39196068
Protein stability (ubiquitination antagonism)	HIF‐1alpha K12la (human)	Non‐histone	Apoptosis	Cross‐species conserved	Sterically blocks VHL recognition, reduces K48 ubiquitination	40760493
Protein stability (ubiquitination antagonism)	RHOA K162la	Non‐histone	Apoptosis	Breast cancer	Antagonizes ubiquitination, stabilizes RHOA (epi‐mutation)	41291745
Protein stability (ubiquitination antagonism)	CCNE2 K347/K348la	Non‐histone	Apoptosis	Hepatocellular carcinoma	SIRT3‐dependent delactylation; impaired delactylation stabilizes CCNE2	36912047
Protein–protein interaction	Vps34 K356/K781la	Non‐histone	Autophagy	Cancer, muscle	Enhances lipid kinase activity via Beclin1/ATG14L/UVRAG binding	37267363
Protein–protein interaction	ALDH2 K52la	Non‐histone	Autophagy (mitophagy)	Acute kidney injury	Disrupts PHB2 interaction, inhibits mitophagy	39737891
Protein–protein interaction	Fis1 K20la	Non‐histone	Apoptosis	Sepsis‐associated AKI	Enhances DRP1 interaction, drives mitochondrial fission	37479690
Protein–protein interaction	Tufm K286la	Non‐histone	Apoptosis	Traumatic brain injury	Disrupts Tomm40 interaction, impairs mitophagy	39496783
Enzymatic activity modulation	METTL3 la (stability)	Non‐histone	Ferroptosis	Intracerebral haemorrhage	Enhances m6A modification of TFRC mRNA, promotes iron uptake	37105375
Enzymatic activity modulation	NSUN2 K508la	Non‐histone	Ferroptosis	Tumour microenvironment	Enhances m5C activity, stabilizes GCLC, suppresses ferroptosis	39742570
Enzymatic activity modulation	HDAC1 K412la	Non‐histone	Ferroptosis	Colorectal cancer	Downregulates FTO/ALKBH5, stabilizes FSP1, suppresses ferroptosis	39888307
Enzymatic activity modulation	AMPKalpha la	Non‐histone	Autophagy	Intervertebral disc degeneration	Inhibits phosphorylation, inactivates AMPK, suppresses autophagy	38570584
Enzymatic activity modulation	p53 K120/K139la	Non‐histone	Apoptosis	Pan‐cancer (AARS1‐dependent)	Reduces DNA binding and LLPS, suppresses pro‐apoptotic transcription	39322678
Subcellular localization	XRCC1 K247la	Non‐histone	Apoptosis	Glioblastoma	Enhances importin alpha binding, nuclear translocation, DNA repair	39111285
Transcriptional regulation	METTL16 K229la / FDX1	Non‐histone	Cuproptosis	Gastric cancer	METTL16 lactylation promotes FDX1 m6A modification, induces cuproptosis	37863889
Protein stability	CIRP la (extracellular)	Non‐histone	PANoptosis	Sepsis	Stabilizes ZBP1, forms PANoptosome with RIPK3, promotes PANoptosis	39465383

Abbreviations: AKI, acute kidney injury; APAP, acetaminophen; ARDS, acute respiratory distress syndrome; EndoMT, endothelial‐to‐mesenchymal transition; I/R, ischaemia–reperfusion; la, lactylation; LLPS, liquid‐liquid phase separation; m5C, 5‐methylcytosine; m6A, N6‐methyladenosine; PCD, programmed cell death.

Collectively, the evidence reviewed above raises the possibility that lactylation functions not merely as a modulator of individual PCD pathways, but as a metabolic–epigenetic node that integrates signals across distinct cell death modalities. A single modification can produce opposing outcomes depending on context. H3K18la promotes pyroptosis in ischaemia–reperfusion injury and neurodegeneration,^69^ yet suppresses it in ulcerative colitis.^72^ H3K18la‐driven transcriptional programmes inhibit ferroptosis in tumours while enhancing it in severe acute pancreatitis.^81^ These bidirectional effects are reinforced by crosstalk among lactylation, acetylation, and ubiquitination at shared lysine residues. They are further amplified by positive feedback loops in which lactylation‐driven upregulation of glycolytic enzymes sustains the modified state. Such features are consistent with switch‐like behaviour, and the concept of lactylation as a cell‐fate rheostat has been discussed in recent reviews.^137^

## 5 CROSS‐TALK BETWEEN LACTYLATION AND OTHER PTMS ON PCD IN HUMAN DISEASES

### 5.1 Lactylation and acetylation

Acetylation, primarily catalysed by acetyltransferases and removed by deacetylases, is a fundamental post‐translational modification that regulates chromatin accessibility, transcriptional activity and protein stability.^138^ Lactylation and acetylation both modify the epsilon‐amino group of lysine, making their occupancy of a single residue mutually exclusive. This competition is reinforced by their shared enzymatic machinery. p300/CBP functions as both an acetyltransferase and a lactyltransferase, while class I histone deacetylases HDAC1‐3 possess both deacetylase and delactylase activity.^139^ The balance between Kla and Kac at a given site therefore depends not only on the relative abundance of acetyl‐CoA and lactyl‐CoA, but also on the metabolic‐state‐dependent catalytic preference of p300 and HDACs. Under basal conditions, intracellular lactyl‐CoA concentrations are 20‐ to 350‐fold lower than acetyl‐CoA levels,^56^ favouring p300‐mediated acetylation. During elevated glycolysis, two mechanisms overcome this constraint. GTPSCS and ACSS2 act as nuclear lactyl‐CoA synthetases, supplying locally enriched lactyl‐CoA to p300/CBP.^25^, ^57^ More critically, HDAC1‐3 can directly catalyse lysine lactylation using free lactate rather than lactyl‐CoA, with a Km of approximately 49µM that falls well within the physiological lactate range of 0.5–20 mM. Triple knockdown of HDAC1‐3 nearly abolishes global Kla, whereas knockdown of p300, CBP or HBO1 has little effect.^59^ HDAC inhibitors decrease Kla while simultaneously increasing Kac,^59^ providing direct pharmacological evidence that these two modifications compete for the same lysine residues.

At the level of individual residues, p53 K120 offers a functionally decisive example of Kla‐Kac antagonism. AARS1 catalyses p53 K120 lactylation in an ATP‐dependent, CoA‐independent manner, directly counteracting p300‐mediated K120 acetylation. K120la reduces p53 DNA‐binding affinity and suppresses liquid‐liquid phase separation, silencing the pro‐apoptotic genes BAX and PUMA and thereby blocking apoptosis.^55^, ^140^ K120ac, in contrast, enhances sequence‐specific DNA binding to promote apoptosis. A single lysine residue thus mediates opposing cell‐death outcomes depending on whether it carries a lactyl or an acetyl group. At the histone level, recombinant HDAC2 catalyses lactylation at H3K9, K14, K18, K23 and K27, all of which are well‐characterized acetylation sites,^59^ indicating that Kla‐Kac competition on histones is systematic. In colorectal cancer, HDAC inhibition increases histone acetylation while concomitantly reducing HDAC1 lactylation, leading to transcriptional activation of m^6^A erasers and sensitization to ferroptosis.^93^ In addition, acetylation can also act as an upstream regulator of Kla by reshaping cellular metabolic states and lactate availability. For instance, hyperacetylation of PDHA1 suppresses pyruvate dehydrogenase activity, promotes lactate accumulation, and subsequently enhances Fis1 lactylation, thereby facilitating mitochondrial dysfunction and apoptosis.^125^ These findings suggest that acetylation and lactylation may exert either cooperative or antagonistic effects on shared substrates depending on cellular context. Overall, the crosstalk between acetylation and lactylation operates at both metabolic and epigenetic levels, providing a mechanistic link between energy metabolism and PCD regulation.

### 5.2 Lactylation and ubiquitination

Ubiquitination represents a central protein quality‐control mechanism that determines protein stability and signaling outcomes through proteasome‐dependent degradation. This modification is dynamically regulated by E3 ubiquitin ligases and deubiquitinases, allowing precise control over cell fate‐associated pathways.^141^

Lactylation‐ubiquitination crosstalk operates primarily through competitive site occupancy: once a lysine is lactylated, ubiquitin cannot be conjugated to the same position, blocking proteasomal degradation. TFEB exemplifies this mechanism. Lactylation at K91, the sole lactylation site on TFEB, prevents binding of the E3 ubiquitin ligase WWP2. This inhibits TFEB ubiquitination and degradation, leading to TFEB accumulation and enhanced autophagy‐lysosomal activity. Elevated TFEB K91la has been detected in human pancreatic ductal adenocarcinoma specimens.^106^ HIF‐1‐alpha follows a similar pattern: lactylation at K12 (human) or K644 (mouse) sterically hinders VHL recognition, reducing K48‐linked polyubiquitination without affecting prolyl hydroxylation. This establishes a metabolic regulatory layer that operates independently of the canonical PHD‐VHL oxygen‐sensing pathway.^142^ Similarly, in cervical cancer, lactylation of DCBLD1 antagonizes its ubiquitination, stabilizes DCBLD1 protein and suppresses apoptosis through metabolic reprogramming.^105^

RHOA lactylation further illustrates the oncogenic potential of this competitive mechanism. K162 lactylation antagonizes ubiquitination to stabilize RHOA, while K118 lactylation impairs its intrinsic GTPase activity, sustaining activation. Together, these two site‐specific modifications functionally recapitulate oncogenic mutations, a phenomenon the authors term reversible epi‐mutation.^143^ Beyond direct site occupancy, lactylation also reshapes the ubiquitination network indirectly. NEDD4 lactylation at K33 reduces its binding to caspase‐11, suppressing caspase‐11 ubiquitination and promoting non‐canonical pyroptosis.^144^ In acetaminophen‐induced liver injury, lactylation of NEDD4 inhibits Caspase‐11 ubiquitination and is associated with enhanced non‐canonical pyroptosis.^75^ In endometrial carcinoma, histone lactylation upregulates the deubiquitinase USP39, which stabilizes PGK1 through deubiquitination and suppresses apoptosis.^119^ Overall, lactylation and ubiquitination engage in crosstalk by regulating protein stability and ubiquitin signaling, thereby linking metabolic reprogramming to the control of PCD.

### 5.3 Lactylation and phosphorylation

Phosphorylation is a rapid and reversible post‐translational modification that plays a central role in signal transduction, metabolic regulation and cell fate determination.^145^ Unlike the preceding crosstalk modes, lactylation and phosphorylation rarely compete for the same residue, because lactylation targets lysine whereas phosphorylation primarily modifies serine, threonine and tyrosine. Their interplay is therefore predominantly indirect. One major route involves phosphorylation‐dependent regulation of lactylation enzymes. ERK‐mediated phosphorylation of ACSS2 at S267 triggers its nuclear translocation and assembly with KAT2A, driving histone lactylation.^25^ In cancer cells, ULK1‐dependent phosphorylation of LDHA enhances lactate production, which promotes Vps34 lactylation and activates autophagy signaling.^107^ A second route involves lactylation‐driven conformational changes that facilitate phosphorylation. Twist1 K150 lactylation promotes its subsequent phosphorylation and nuclear translocation, driving endothelial‐to‐mesenchymal transition.^146^ In Alzheimer disease brains, tau K331 lactylation enhances tau hyperphosphorylation.^147^ In vascular inflammation, PI3K/AKT‐dependent phosphorylation enables Sox10 lactylation, which enhances its transcriptional activity and promotes non‐canonical pyroptosis during vascular smooth muscle cell transdifferentiation.^76^ In intervertebral disc degeneration, glutamine suppresses glycolysis‐driven AMPKα lactylation while enhancing AMPKα phosphorylation, thereby activating autophagy.^110^ Collectively, these studies reveal that the crosstalk between lactylation and phosphorylation operates through both cooperative and antagonistic modes.

## 6 SMALL MOLECULAR COMPOUNDS TARGETING LACTYLATION‐MODIFIED PCD IN HUMAN DISEASES

### 6.1 Targeting glucose uptake and lactate production

Lactylation depends on intracellular lactate availability. Strategies that limit glucose uptake or glycolytic flux therefore reduce the substrate pool available for this modification. STF‐31, WZB‐117 and BAY‐876 are GLUT1 inhibitors with demonstrated anti‐tumour activity in renal cell carcinoma and ovarian cancer models.^148^, ^149^, ^150^ The glycolytic inhibitor 2‐deoxy‐D‐glucose (2‐DG) and oxalate suppress H3K18 lactylation and enhance CD8‐positive T‐cell cytotoxicity in non‐small cell lung cancer.^151^ Stiripentol, a clinically approved LDHA inhibitor originally developed for epilepsy, reduces intracellular lactate and inhibits NBS1 K388 lactylation, attenuating DNA repair‐associated chemoresistance in gastric cancer.^152^ Other LDHA inhibitors, including GSK2837808A and FX11, have shown preclinical efficacy in restricting lactate output and lactylation‐dependent phenotypes, though none have entered oncology trials. Despite their mechanistic appeal, glucose‐ and lactate‐targeting agents face a narrow therapeutic window, because normal tissues with high glycolytic demand, including the brain and skeletal muscle, depend on the same pathways.

### 6.2 Targeting lactate transport

Blocking lactate flux across the plasma membrane offers an alternative to targeting intracellular production. Monocarboxylate transporter 1 (MCT1) mediates lactate uptake in oxidative tumour cells; MCT4 facilitates lactate efflux from glycolytic cells. alpha‐Cyano‐4‐hydroxycinnamate (CHC), an MCT1 inhibitor, attenuates lactate‐induced Snail1 lactylation and reduces myocardial fibrosis after infarction.^153^ Syrosingopine and lonidamine, which target MCT4, disrupt lactate shuttling in preclinical models.^154^ The most clinically advanced agent in this class is AZD3965, a selective oral MCT1 inhibitor. It was evaluated in a multicentre phase I dose‐escalation trial in patients with advanced solid tumours and non‐Hodgkin lymphoma, where it demonstrated acceptable safety with on‐target, reversible ocular toxicities and achieved target engagement at the recommended phase II dose of 10 mg twice daily.^155^ This trial provides the first clinical proof of concept for pharmacological MCT1 blockade and represents the most advanced clinical‐stage strategy for indirectly modulating lactylation in oncology. Patient selection guided by tumour MCT1/MCT4 expression status may further improve the therapeutic index.

### 6.3 Targeting lactylation writers and erasers

Directly inhibiting the enzymes that deposit or remove lactyl groups offers a more proximal route to modulating lactylation‐dependent PCD. p300, a histone acetyltransferase with lactylation writer activity, has been targeted by several compounds. A‐485, a selective p300 catalytic inhibitor, suppresses YY1 lactylation and pathological angiogenesis in ocular neovascularization models.^156^ Andrographolide, a natural product, inhibits histone lactylation by targeting p300 and alleviates aortic valve calcification.^157^ C646, another p300 inhibitor, shows enhanced anti‐tumour activity when combined with the BRAF inhibitor vemurafenib.^158^ Beyond p300, HDAC1‐3 are now recognized as bifunctional enzymes that remove and catalyse lysine lactylation depending on metabolic context.^59^ Several HDAC inhibitors, including vorinostat (SAHA) and entinostat (MS‐275), are already approved for haematological malignancies. Their ability to shift the Kla‐Kac balance at shared lysine residues raises the possibility of repurposing these agents for lactylation‐directed therapy. Pharmacological HDAC inhibition decreases global Kla while increasing Kac, an effect exploited to sensitize colorectal cancer cells to ferroptosis.^59^ The broad substrate specificity of both p300 and HDAC inhibitors, however, complicates the attribution of therapeutic effects specifically to lactylation modulation.

### 6.4 Emerging strategies and clinical outlook

Beyond small molecules, several emerging approaches extend the therapeutic landscape. Cold atmospheric plasma (CAP) modulates H3K18 lactylation via the USP49/HDAC3 axis, driving ferroptosis in endometrial cancer models.^6^ The CAPmed‐BC database provides integrated multi‐omics resources for systematic analysis of CAP‐induced lactylation dynamics.^159^ Lactylation also intersects with cancer immunotherapy. H3K18la‐driven PD‐L1 expression promotes immune evasion in ovarian cancer and non‐small cell lung cancer.^160^ Pharmacological inhibition of lactylation restores immune checkpoint inhibitor sensitivity in hepatocellular carcinoma models.^161^ These findings support evaluating lactylation‐targeted agents in combination with immunotherapy. Several barriers nonetheless remain. The therapeutic index of glycolytic inhibitors is constrained by the reliance of normal tissues on glucose metabolism. The redundancy of lactylation writers and incomplete understanding of substrate specificity complicate target selection. No validated biomarker exists for patient stratification in lactylation‐directed trials. Addressing these gaps, particularly through isoform‐selective inhibitors and companion biomarkers, will be essential for translating the preclinical rationale into clinical benefit.

## 7 POTENTIAL THERAPEUTIC APPLICATIONS OF LACTYLATION‐MODIFIED PCD IN HUMAN DISEASES

Recent advances suggest that integrating lactylation with PCD regulation may provide innovative solutions to the challenges faced by conventional anti‐cancer strategies. Various PCD and lactylation‐related regulators have been implicated in modulating tumour cell sensitivity to chemotherapy or, playing critical roles in the development of drug resistance (Table 2).

**TABLE 2:** Potential therapeutic applications of lactylation‐modified programmed cell death in human diseases.

Lactylation site	Target	PCD type	Disease	Therapeutic resistance context	Mechanism	References
XRCC1 K247	XRCC1/importin α	Apoptosis	Glioblastoma (GBM)	Radiotherapy and chemotherapy resistance	XRCC1 K247 lactylation increases binding to importin α, promotes nuclear import and DNA repair, reduces therapy‐induced apoptosis, and thereby enhances resistance to radiotherapy and chemotherapy.	^123^
H3K18la	circATXN7/NF‐κB p65	Apoptosis	Colorectal cancer	Immunotherapeutic resistance	H3K18la‐associated circATXN7 regulation modulates NF‐κB p65 signaling and affects activation‐induced cell death of tumour‐specific T cells, thereby influencing the response to immunotherapy.	^118^
NBS1 K388	MRN complex	Apoptosis	Gastric cancer	Chemotherapy resistance	NBS1 K388 lactylation supports MRN complex activity, enhances DNA repair capacity, and reduces chemotherapy‐induced apoptosis.	^53^
IGF‐1R	MET/CDKN2A axis	Cuproptosis	Multiple myeloma	Proteasome inhibitor resistance	IGF‐1R lactylation activates MET signaling and upregulates CDKN2A, suppressing FDX1‐associated cuproptosis and promoting resistance to proteasome inhibitors.	^127^
H3K18la	ZFP64/GCH1/FTH1	Ferroptosis	Breast cancer	Doxorubicin resistance	H3K18la‐induced ZFP64 expression activates GCH1 and FTH1, limiting lipid peroxidation and intracellular Fe2+ accumulation and thereby contributing to doxorubicin resistance.	^78^
H3K18la	RUBCNL/BECN1	Autophagy	Colorectal cancer	Bevacizumab resistance	H3K18la‐driven RUBCNL expression promotes interaction with BECN1 and facilitates autophagosome maturation, thereby enhancing bevacizumab resistance.	^92^

Abbreviations: DOX, doxorubicin; GBM, glioblastoma; MRN, MRE11–RAD50–NBS1; PCD, programmed cell death.

### 7.1 Chemotherapy and radiotherapy resistance

Lactylation‐modulated apoptosis is a major mechanism by which tumour cells evade genotoxic therapy. XRCC1 lactylation at K247 enhances its affinity for importin alpha, promoting nuclear translocation and DNA repair in glioblastoma and reducing chemo‐radiotherapy‐induced apoptosis.^162^ NBS1 lactylation at K388 augments DNA repair through the MRE11‐RAD50‐NBS1 (MRN) complex, attenuating chemotherapy‐induced apoptosis in gastric cancer.^163^ A common theme emerges: lactylation stabilizes or relocalizes DNA repair factors, blunting the apoptotic response to DNA‐damaging agents. SIRT3‐dependent delactylation of CCNE2 at K347 and K348 suppresses hepatocellular carcinoma growth,^164^ implying that impaired delactylation in tumours with low SIRT3 expression may contribute to intrinsic chemoresistance. Beyond DNA repair, lactylation sustains anti‐apoptotic signalling through epigenetic routes. In endometrial carcinoma, H3K18la‐driven upregulation of USP39 stabilizes PGK1 and activates the PI3K/AKT pathway, suppressing apoptosis^165^.

### 7.2 Targeted therapy and immunotherapy resistance

Lactylation also underpins resistance to molecularly targeted agents and immunotherapies. ZFP64 upregulation driven by H3K18la promotes GCH1‐mediated lipid peroxidation inhibition and FTH1‐dependent iron sequestration, conferring doxorubicin resistance in triple‐negative breast cancer.^166^ IGF‐1R lactylation stabilizes the receptor and activates MET signalling, upregulating CDKN2A and suppressing FDX1 to drive resistance to proteasome inhibitors in multiple myeloma.^167^ H3K18la‐driven RUBCNL expression enhances autophagosome maturation through BECN1 interaction, contributing to bevacizumab resistance in colorectal cancer.^168^ Lactylation also intersects with immunotherapy resistance. H3K18la‐driven PD‐L1 expression promotes immune evasion in ovarian cancer and non‐small cell lung cancer.^160^ Pharmacological inhibition of lactylation restores immune checkpoint inhibitor sensitivity in hepatocellular carcinoma models.^161^. CircATXN7 lactylation sensitizes tumour‐specific T cells to activation‐induced cell death through NF‐kappaB signalling, promoting resistance to immunotherapy in colorectal cancer.^169^

### 7.3 Emerging physical modalities

Beyond pharmacological interventions discussed in Section 6, physical modalities have begun to emerge as alternative routes for modulating lactylation. Histone lactylation has been proposed as an epigenetic mark of the glycolytic switch, framing metabolic reprogramming as a targetable vulnerability.^170^ Cold atmospheric plasma (CAP), a partially ionized gas at near‐ambient temperature, can modulate this switch. In endometrial cancer, CAP suppresses HDAC3‐mediated H3K18 lactylation through the USP49/HDAC3 axis, reinforcing p53 expression and triggering ferroptosis in vitro and in xenograft models.^6^ The CAPmed‐BC database was recently established as the first multi‐omics platform in plasma medicine, integrating lactylome, acetylome, proteome, phosphoproteome and transcriptome data from CAP‐treated breast cancer cells.^159^ These developments suggest that physical plasma technologies may complement pharmacological inhibitors in targeting the lactylation‐PCD axis, although clinical translation remains at an early stage.

### 7.4 Translational perspectives

The diversity of lactylation‐dependent resistance mechanisms suggests that a single‐agent approach targeting one PCD pathway may be insufficient, as tumours can engage compensatory cell death programmes. Combination strategies that pair lactylation‐targeted agents with conventional therapies warrant systematic preclinical evaluation. Several priorities emerge. First, the development of isoform‐specific lactylation inhibitors with favourable pharmacokinetic profiles is needed to enable chronic dosing. Second, pharmacodynamic biomarkers, such as circulating lactylated proteins or imaging‐based assessments of tumour lactate, must be validated for patient stratification. Third, the therapeutic index of lactate‐lowering strategies requires careful assessment, given the reliance of normal tissues on lactate as a metabolic fuel and signalling molecule. Addressing these priorities will determine whether the preclinical rationale for targeting lactylation‐modified PCD translates into meaningful clinical benefit.

## 8 CONCLUSIONS AND PERSPECTIVES

In recent years, lysine lactylation has emerged as a novel PTM that has garnered increasing attention for its role in PCDs. Studies have demonstrated that lactate is not merely the end product of glycolysis, but also functions as a critical signaling molecule. By modulating both histone and non‐histone lactylation, lactate participates in multiple forms of PCD, including apoptosis, autophagy, ferroptosis and pyroptosis, thereby exerting key regulatory effects in various human diseases such as cancer, neurodegenerative disorders, ischaemia–reperfusion injury and infectious diseases. This modification influences epigenetic regulation, metabolic reprogramming, oxidative stress responses and immune microenvironment remodelling, underscoring its role as a molecular bridge between metabolic signalling and cell fate determination.

Although current research has begun to elucidate the functional significance of lactylation in PCD regulation, numerous scientific challenges remain. For example, its enzymatic mechanism remains poorly defined, and no universally accepted ‘writers’ or ‘erasers’ have been identified, limiting a comprehensive understanding of its regulatory network. Moreover, it is still unclear whether lactylation arises passively in response to elevated lactate levels or represents a spatiotemporally regulated process involving specific enzymatic activity. The regulatory nature of this modification has yet to be conclusively determined. In addition, the potential metabolic substrates of lactylation—such as lactoyl‐CoA and lactoyl‐glutathione—have not been systematically characterized in terms of their intracellular dynamics, metabolic origins and functional roles in the lactylation process. Furthermore, lactylation may interact with or compete against other lysine‐targeted PTMs—such as acetylation, methylation and ubiquitination—for shared modification sites, or act in synergy or antagonism to modulate protein function. However, the precise interaction patterns among these modifications, particularly within different types of PCD, remain largely undefined and warrant further investigation. Looking ahead, with the rapid advancement of multi‐omics technologies such as proteomics, metabolomics, and spatial omics, research on lactylation is expected to progress from phenotypic characterization to mechanistic elucidation. Future studies are likely to focus more on the spatiotemporal dynamics and biological functions of this modification, providing novel molecular insights into the pathogenesis of complex diseases and offering promising targets for precision medical interventions.

Future research should systematically focus on several key directions. First, it is crucial to identify and clarify the specific regulatory pathways and key targets of lactylation in various types of PCD, elucidating its mechanistic roles in apoptosis, autophagy, ferroptosis, pyroptosis and other forms of PCD. Second, advanced multi‐omics technologies should be integrated and applied to dynamically monitor the spatiotemporal changes of lactylation during disease onset and progression, thereby revealing its regulatory networks and functional relationships. In addition, targeted efforts are needed to develop specific small‐molecule inhibitors or activators, monoclonal antibodies, and gene‐editing tools to precisely modulate key enzymes and signalling pathways involved in lactylation, enabling precise intervention and therapy in pathological processes. Meanwhile, potential physiological side effects resulting from lactylation modulation require careful consideration. It remains unclear whether long‐term regulation of lactate metabolism or transport disrupts metabolic homeostasis and the balance of PCD in normal cells, which could affect therapeutic safety and efficacy. Therefore, large‐scale, multicenter and mechanistically well‐defined preclinical and translational studies are urgently needed to systematically evaluate the regulatory roles of lactylation in PCD and disease progression, as well as to assess its feasibility, safety and clinical potential as a diagnostic biomarker and therapeutic target. Future in‐depth research will not only advance the fundamental understanding of lactylation but also provide novel concepts and technical support for precise diagnosis and personalized treatment of complex diseases. This will promote the interdisciplinary integration of metabolic regulation and cell fate research, ultimately facilitating breakthroughs from molecular mechanisms to clinical translation.

In summary, recent advances in the study of protein lactylation and PCD have deepened our understanding of the molecular mechanisms involved in disease pathogenesis. Lactylation has emerged as a crucial regulator of various forms of PCD across multiple diseases, highlighting its potential as a promising therapeutic target. To this end, we aim to further elucidate the precise mechanisms by which lactylation modulates different PCD pathways. Moreover, we expect an increasing number of clinical studies to explore the interplay between lactylation and PCD. Ultimately, clarifying this complex relationship may facilitate the development of novel therapeutic approaches and improve patient outcomes.

## AUTHOR CONTRIBUTIONS

*Conceptualization, funding acquisition, and supervision*: Hengwei Liu, Yi Liu and Yue Chen. *Roles/writing—original draft*: Yue Chen and Xiwen Wen. *Writing—review and editing*: All authors. All co‐authors contributed to subsequent drafts of the manuscript, and all authors have seen and approved the final version.

## CONFLICT OF INTEREST STATEMENT

The authors declare no conflicts of interest.

## ETHICS STATEMENT

Ethical approval was not required for this review, as all data were derived from previously published studies and no new studies involving human participants or animals were conducted.

## Data Availability

No datasets were generated or analysed during the current study.
